# Enumerating all maximal frequent subtrees in collections of phylogenetic trees

**DOI:** 10.1186/1748-7188-9-16

**Published:** 2014-06-18

**Authors:** Akshay Deepak, David Fernández-Baca

**Affiliations:** 1 Department of Electrical and Computer Engineering, Iowa State University, Ames, Iowa, USA; 2 Department of Computer Science, Iowa State University, Ames, Iowa, USA

**Keywords:** Phylogenetic trees, Evolutionary trees, Maximum agreement subtree, Frequent subtrees, Maximal frequent subtrees, Reverse search

## Abstract

**Background:**

A common problem in phylogenetic analysis is to identify frequent patterns in a collection of phylogenetic trees. The goal is, roughly, to find a subset of the species (taxa) on which all or some significant subset of the trees agree. One popular method to do so is through maximum agreement subtrees (MASTs). MASTs are also used, among other things, as a metric for comparing phylogenetic trees, computing congruence indices and to identify horizontal gene transfer events.

**Results:**

We give algorithms and experimental results for two approaches to identify common patterns in a collection of phylogenetic trees, one based on agreement subtrees, called maximal agreement subtrees, the other on frequent subtrees, called maximal frequent subtrees. These approaches can return subtrees on larger sets of taxa than MASTs, and can reveal new common phylogenetic relationships not present in either MASTs or the majority rule tree (a popular consensus method). Our current implementation is available on the web at https://code.google.com/p/mfst-miner/.

**Conclusions:**

Our computational results confirm that maximal agreement subtrees and all maximal frequent subtrees can reveal a more complete phylogenetic picture of the common patterns in collections of phylogenetic trees than maximum agreement subtrees; they are also often more resolved than the majority rule tree. Further, our experiments show that enumerating maximal frequent subtrees is considerably more practical than enumerating ordinary (not necessarily maximal) frequent subtrees.

## Background

A phylogenetic tree is an unordered rooted tree whose leaves are in one-to-one correspondence with a set of species (also referred to as taxa); its topology represents the hypothetical evolutionary relationships among these species.

An *agreement subtree* (AST) for a collection of phylogenetic trees on a common leaf set is a minimal subtree connecting a fixed set of leaves that is homeomorphically included in all of the input trees. A *maximal agreement subtree* (MXST) is an agreement subtree that is not a subtree of any other agreement subtree. An MXST is a *maximum agreement subtree* (MAST) if it has the largest number of leaves [1]. MASTs are used, among other things, as a metric for comparing phylogenetic trees [2-4], computing their congruence index [5,6], to identify horizontal gene transfer events [7], for resolving ambiguity in terraces in phylogenetic tree space [8], and as a consensus approach [9].

**Figure 1:**
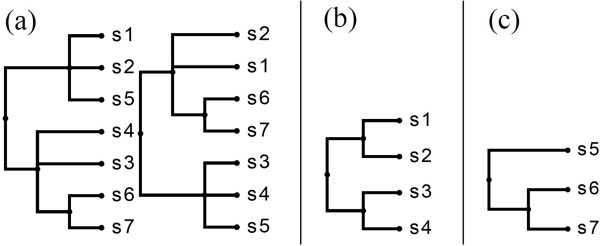
**Motivating example 1.(a)** A collection of two trees and their **(b)** MAST. **(c)** An MXST that has fewer leaves than the MAST but is not displayed by it.

An MXST can reveal shared phylogenetic information not displayed by any of the MASTs (see Figure 1). We can uncover even more common substructure by relaxing the requirement that the subtree returned must be supported by all the input trees. Let *f* be a number in the interval $\left(\frac{1}{2},1\right]$. An *f*-frequent subtree, or a *frequent subtree* (FST) for short, in a collection of *m* leaf-labeled trees on a common leaf set, is a minimal subtree connecting a fixed set of leaves that is homeomorphically included in at least *f*·*m* of the input trees. A *maximal FST* (MFST) is an FST that is not a subtree of any other FST. We choose *f* greater than $\frac{1}{2}$, because (i) it conveys confidence that a majority of the input trees support the *f*-frequent subtree, and (ii) it ensures uniqueness: on a given set of leaves, there can be at most one *f*-frequent subtree. Observe that an MXST is an MFST with *f*=1.

The set of all MFSTs is a compact non-redundant summary of the set of all FSTs: every FST is a subtree of one or more MFSTs but every MFST is a subtree of only itself. Thus, every MFST reveals some unique phylogenetic information that is not displayed by any other MFST (or FST).

**Figure 2:**
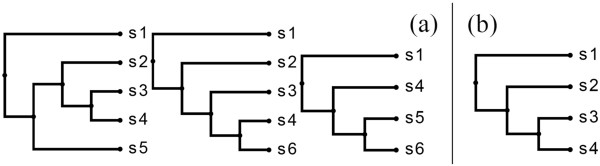
**Motivating example 2.(a)** A collection of three trees and **(b)** an MFST with $f=\frac{2}{3}$. MAST or MRT cannot be applied, as the common overlap consists of only two leaves.

Also, since there can be exponentially more FSTs than MFSTs, mining MFSTs can be much faster than mining all FSTs, and the result set produced is much smaller and easier to analyze.A well-supported MFST can have more leaves and be more resolved than a MAST (see “Results and discussion” on page 14), and thus can reveal phylogenetic information not displayed by any of the MASTs. In the more general setting where there is little overlap among the leaf sets of the input trees, the gap between the size of an MFST and the size of a MAST can be even wider. Indeed, in this case any agreement tree would tend to be quite small — see Figure 2.

Despite its potential utility, however, the enumeration of all MFSTs in collections of phylogenetic trees has not, to our knowledge, been studied before.

Here we introduce MFSTMINER, an algorithm for enumerating MXSTs and MFSTs. MFSTMINER enumerates MFSTs over partially overlapping leaf sets as well. We compare MFSTMINER with EVOMINER[10], an algorithm for enumerating all FSTs, and show that enumerating MFSTs can be orders of magnitude faster than enumerating all FSTs. Our current implementation of MFSTMINER, which works for up to 250 leaves and 10000 trees, can be downloaded from https://code.google.com/p/mfst-miner/.

### Related work

The MAST problem was first studied by Finden and Gordon [1]. Since then, due its utility and inherent complexity, the problem has attracted computational biologists and mathematicians alike. The MAST of two trees can be found in polynomial time; indeed, over the years researchers have developed progressively faster algorithms for the problem [11-13]. Finding the MAST of more than two trees is NP-hard in general [11], but is solvable in polynomial time for trees of bounded degree [14,15].

Although maximal subgraph mining [16,17] and, in particular, maximal subtree mining [18-20] have received much attention in the data mining literature, a different approach is needed for mining phylogenetic trees. This is because phylogenetic trees possess a special structure — only leaves are labeled and the non-leaf nodes must be of degree two or more — that affects the very definition of a subtree [10,21]. We defer the formal definitions of phylogenetic trees and subtrees to the Preliminaries, on page 4.

Zhang et al. [21] where the first to study frequent phylogenetic subtree mining. They proposed an algorithm, Phylominer, to mine all frequent subtrees in a collection of phylogenetic trees in time quadratic in the size of the output set. In [10] we proposed a new algorithm EVOMINER for the same task that achieves speed-ups of up to 100 times or more over Phylominer. Both Phylominer and EVOMINER follow an Apriori-like framework [22]. EVOMINER’s increased speed is the result of an efficient phylogenetic tree-specific constant-time candidate generation scheme in the candidate generation step, and a novel fingerprinting based scheme for the downward-closure operation in the frequency counting step.

Ramu et al. [23] proposed a heuristic for enumerating a subset of all MFSTs called maximum frequent subtrees. A maximum frequent subtree is an FST that has the maximum number of leaves among all FSTs. Their method scales well for large phylogenetic datasets, but does not guarantee the enumeration of all MFSTs. To our knowledge, our work is the first to deal with the problem of mining all MFSTs for phylogenetic trees.

Consensus methods are an oft-used alternative to frequent or agreement subtree methods, for summarizing the common information in collections of phylogenetic trees. Among the most popular consensus methods is the *majority-rule tree* (MRT) [24], defined as follows.

**Figure 3:**
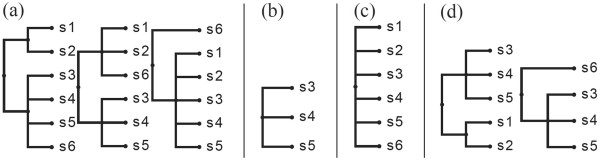
**Motivating example 3.(a)** Three input trees. **(b)** Their MAST, which is star-like. **(c)** The majority rule tree, which is also star-like. **(d)** Two MFSTs with $f=\frac{2}{3}$, each highly resolved and larger than the MAST.

A *cluster* in a tree is the set of all leaf descendants of some node in the tree. The MRT of a collection of trees is the tree that exhibits all clusters present in the majority —i.e., strictly more than 50%— of the input trees. (Note the parallels between the use of majority clusters and the choice of $f\in \left(\frac{1}{2},1\right]$ for MFSTs.) The MRT, though linear-time computable, is very sensitive to the presence of “rogue” taxa; that is, taxa whose positions vary widely within the input collection [25,26]. MFSTs are less sensitive to this phenomenon, because the MRT by definition must contain the entire leaf set (including the rogue taxa), whereas MFSTs have no such restriction (see Figure 3). The fact that MASTs are less sensitive to rogue taxa than MRTs has been well-acknowledged in the literature [25,27,28]. MFSTs, which include MASTs as a special case, are even more likely to reveal informative common substructures in the presence of rogue taxa.

Phylogenetic *networks* represent evolutionary relationships among taxa via directed graphs. In addition to tree nodes —nodes with only one parent—, they allow hybrid nodes —nodes with two parents. Thus, phylogenetic networks are more expressive than phylogenetic trees [29,30]. In the same way that agreement trees and majority rule trees extract consensus information in phylogenetic trees, *consensus networks*[31] represent frequent patterns in phylogenetic networks [32].

### Preliminaries

A ***phylogenetic tree*** (or, for brevity, simply a ***tree***) is an unordered rooted leaf-labeled tree. Leaf labels represent the taxonomic units (species or taxa) under study. An isomorphism between phylogenetic trees includes the labels of the leaves. Phylogenetic trees can also be unrooted [33], but here we deal exclusively with rooted phylogenetic trees. A node is ***internal*** if it is not a leaf node. Each internal node must have at least two children. Let ${ℒ}_{T}$ denote the leaf label set of tree *T*, and *ψ*_*T*_ denote the bijection that maps the leaf nodes to their unique labels. For convenience, we refer to the set of leaf nodes by their labels in ${ℒ}_{T}$. From this point forward, unless the context requires making a distinction, we will drop the subscripts in ${ℒ}_{T}$ and *ψ*_*T*_, and write  and *ψ* respectively. For the rest of the paper, we assume without loss of generality that the leaf label set  consists of distinct integers in the range $\left[1,|ℒ|\right]$; thus, the labels are ordered. We denote the fact that two trees *T*_1_ and *T*_2_ are isomorphic by writing *T*_1_≡*T*_2_.

**Figure 4:**
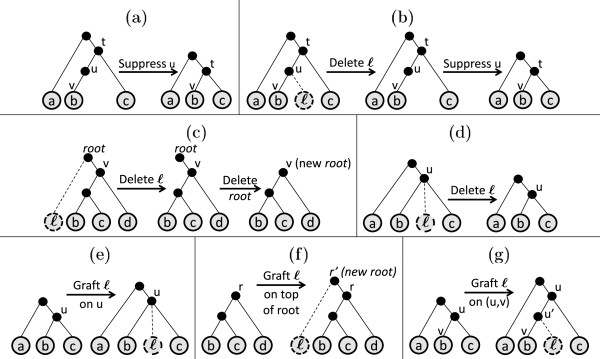
**Pruning in phylogenetic trees.(a)** Suppressing a node. **(b)** Pruning with node suppression. textbf(c) Pruning a leaf attached to a degree-two root. **(d)** Pruning without node suppression. **(e)** Grafting on a non-root node. **(f)** Grafting on root. **(g)** Grafting on an edge.

Let *T* be a tree. Suppose *u* is an internal non-root node in *T*, such that *u* has only one child *v*. Then, *suppressingu* means contracting the edge (*u*,*v*); i.e., deleting *u* and the two edges incident on it, and then adding an edge from the parent of *u* to *v*. For example, in Figure 4(a), to suppress *u*, it is deleted and an edge is added from *t* to *v*. To *prune* a leaf *ℓ*, we first delete it. Let *u* be *ℓ*’s parent. If *u* is not the root, and the deletion of *ℓ* makes *u* a degree-two node, we suppress *u* (see Figure 4(b)). If *u* is the root and deleting *ℓ* makes it a degree one node, *u* is deleted and its remaining child becomes the new root (see Figure 4(c)). Otherwise, *u* remains as it is (see Figure 4(d)). Grafting is the reverse of pruning a leaf. Consider a leaf $\ell \notin {ℒ}_{T}$. To *graftℓ* in *T*, we first select a node or an edge in *T*. If the selection is a non-root node *u*, we make *ℓ* a child of *u* in *T* (see Figure 4(e)). We call this grafting of *ℓ* on node *u*. If the selection is root node *r*, we have two options: (i) graft *ℓ* on *r* as if *r* is a non-root node, or (ii) create a new node *r*’ and make *r* and *l* as children of *r*’. In case (ii), *r*’ becomes the new root in *T* (see Figure 4(f)); we call this *graftingℓon top of root node r*. If the selection is an edge (*u*,*v*), where *u* is the parent of *v*, we delete edge (*u*,*v*), create a new node *u*’, make *u*’ a child of *u*, and, *ℓ* and *v* children of *u*’ (see Figure 4(g)). We call this *grafting ofℓon edge* (*u*,*v*). Let *T*’ denote the resulting tree. Then, clearly, in each of the cases, *T* can be obtained by pruning *ℓ* in *T*’.

Consider a tree *T* and a set ${ℒ}^{\prime }\subseteq {ℒ}_{T}$. The *restriction* of *T* to ${ℒ}^{\prime }$, denoted by $T{|}_{{ℒ}^{\prime }}$, is the minimal homeomorphic subtree of *T* connecting the leaves with labels in ${ℒ}^{\prime }$ (that is, we start with the minimal subtree of *T* connecting ${ℒ}^{\prime }$, and repeatedly suppress non-root nodes with at most one child until no such nodes remain). A tree *T*^′^ is a ***subtree*** of *T* if ${ℒ}_{T^{\prime }}\subseteq {ℒ}_{T}$ and $T^{\prime }\equiv T{|}_{{ℒ}_{T^{\prime }}}$. Tree *T* ***displays*** *T*’ if *T*’ is a subtree of *T*.

The ***depth*** of a node *u* in a tree *T*, denoted depth^*T*^(*u*), is the number of edges from the root to that node; thus the root node is at depth 0. We denote the lowest common ancestor (LCA) of two nodes *u* and *v* in *T* by LCA^*T*^(*u*,*v*). When the tree *T* is clear from the context, we drop the superscripts. A ***k***-leaf tree is a tree with *k* leaves. A ***triplet*** is a 3-leaf tree.

## Algorithmic framework

We first discuss the algorithm for ASTs/MXSTs because it is simpler (since *f*=1). We then extend it to FSTs/MFSTs.

We enumerate all MXSTs from the solution space of all ASTs. We show that any *k*-leaf AST can be enumerated by combining two unique (*k*−1)-leaf ASTs with certain properties. We call the *k*-leaf tree a ***join*** on the two smaller (*k*−1)-leaf trees. To ensure that the enumeration is efficient, we must address three issues. The first is *avoiding redundancy* — that is, each MXST should be generated only once.

The second is *support estimation*. While a *k*-leaf AST is enumerated by joining two unique (*k*−1)-leaf ASTs, the converse is not true, i.e., these two (*k*−1)-leaf ASTs can potentially combine into more than one topology over *k* leaves. The only way to know if a *k*-leaf AST exists as a result of joining these two (*k*−1)-leaf ASTs is to have a mechanism to test if only one topology is supported across all input trees. The third issue is *limiting combinatorial explosion.* The total number of ASTs can be exponentially larger than the total number of MXSTs. Thus, we need a way to prune the search space of ASTs during enumeration of MXSTs. We describe how we address these issues next.

### Non-redundant enumeration

To avoid generating multiple isomorphic copies of the same tree, we enumerate subtrees in “canonical form” [21] (an ordered representation for phylogenetic trees). To enumerate every canonical representation once, we define a parent-child relationship over the space of all ASTs. This induces an enumeration tree over the solution space, where each node represents a collection of ASTs grouped together via an equivalence relation. Leaf nodes represent potential MXSTs and each MXST belongs to a unique leaf node. This scheme is motivated by the reverse search technique for enumeration [34].

#### Canonical form

The ***virtual label*** of an internal node *v* is the minimum label among all leaf descendants of *v*. Consider a left-to-right order on the children of an internal node based on the sequence in which they are encountered in an inorder depth-first traversal (IDFT), the leftmost child being encountered first. Then, a tree *T* is in ***canonical form*** [21] if, for every internal node, its children are ordered from left to right by their virtual labels. It can be seen that two trees are isomorphic if and only if they have the same canonical forms. By generating all trees in canonical form, it is straightforward to test if two trees are isomorphic and prevent duplicate enumeration. MFSTMINER relies on this property to ensure that each FST is enumerated exactly once. Henceforth, we assume all trees to be in canonical form unless mentioned otherwise.

#### Enumeration tree

**Figure 5:**
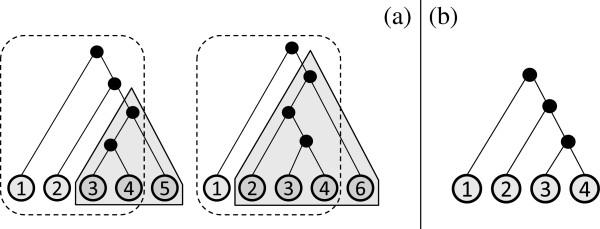
**Equivalence class.(a)** Two trees belonging to the same equivalence class. The common prefix tree (shown separately in **(b)**) is encircled by the dotted lines; the respective rightmost leaves are the ones outside the dotted lines. The shaded part represents the respective heaviest subtrees.

The key notion for defining the enumeration tree is that of an *equivalence class*; to explain it, we first need some definitions. The ***rightmost leaf*** of tree *T* is the last leaf encountered in the IDFT of *T*. The subtree that results from pruning the rightmost leaf is called the ***prefix tree*** or ***prefix*** for short. It is so called because the IDFT of the prefix tree is the largest prefix of the IDFT of the original tree that is not the original tree. A useful property of the canonical form is that pruning either the last or second-to-last leaf encountered in the IDFT of a tree results in a canonical tree [21]. The ***heaviest subtree*** [21] is the subtree rooted at the parent of the rightmost leaf. Figure 5 illustrates the defined concepts.

An ***equivalence class*** is a set *E* of canonical trees that share a common prefix. We call this common prefix tree the ***core tree*** of *E* and denote it by *E*^*c*^. Note that an equivalence class of *k*-leaf trees has a (*k*−1)-leaf core tree. For an AST *T*, $E_{T}$ denotes the equivalence class that has *T* as its core tree. Any two trees in an equivalence class differ only with respect to their rightmost leaf; therefore, topologically, their difference is restricted to their heaviest subtrees. The equivalence relation “sharing a common prefix” partitions any set of canonical trees into disjoint subsets. Each such subset is an equivalence class identified by its unique core tree.

**Figure 6:**
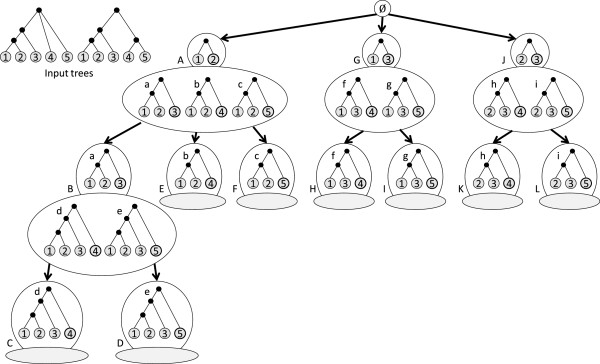
**Enumeration Tree example.** Each node in the tree represents an equivalence class. Trees in an equivalence class differ only with respect to their rightmost leaves (circled in bold for each tree). The bubble at the top of a node contains the core tree of the corresponding equivalence class. An equivalence class contains all ASTs that have its core tree as their common prefix. The core tree of an equivalence class belongs to its parent equivalence class. For example, the core tree of equivalence class *B* is *a*, which belongs to *A* — the parent of *B*. All 3-leaf ASTs have been partitioned into equivalence classes *A*, *G* and *J* (children of the root node). The leaf nodes (indicated by shaded ellipses) are empty equivalence classes and their core trees represent potential MXSTs. Here, *d* and *e*, the respective core trees of leaf nodes *C* and *D*, are the only MXSTs. They also happen to be the MASTs for the input trees.

Figure 6 gives an example of an enumeration tree. Each node in the enumeration tree represents a unique equivalence class. An equivalence class *E* is the *parent* of equivalence class *F* if *F*^*c*^∈*E*. Clearly each node has a unique parent. Note that as we traverse from an internal node towards a leaf, the core tree of each node in the path corresponds to a new leaf being added as a suffix to the IDFT of the core tree of its parent node. Thus, if equivalence class *E* is an ancestor of equivalence class *F*, the IDFT of *E*^*c*^ is a prefix of the IDFT of *F*^*c*^.

A node in the enumeration tree is a *leaf* if its core tree is not a prefix to any other AST. Thus, its corresponding equivalence class is empty. Note that every MXST is the core tree of some leaf node. The converse is not true, because a (*k*−1)-leaf tree may not be the prefix of a given *k*-leaf tree, yet can be a subtree of it. The root of the enumeration tree is an empty node that has all the equivalence classes containing 3-leaf ASTs as its children. This is because three is the minimum number of leaves on which phylogenetic inference can be meaningful. For an equivalence class *E*, the *branch* at *E* represents the subtree induced in the enumeration tree by all the leaf descendants of *E*, or simply *E* if it is a leaf. ASTs *X* and *Y* are considered to be of a *common descent* if neither is a descendant of the other.

#### Pairwise join

The canonical form has the property that pruning either the last leaf or the second-to-last leaf encountered in the IDFT yields a subtree that is also canonical [21]. Thus, every *k*-leaf AST *T* corresponds to a unique ordered pair (*T*_*x*_,*T*_*y*_) of (*k*−1)-leaf ASTs where *T*_*x*_ and *T*_*y*_ are obtained by pruning the last leaf and the second-to-last leaf respectively in the IDFT of *T*. Note that *T*_*x*_ and *T*_*y*_ share a common prefix. Conversely, *T* can be obtained by “joining” this unique pair (*T*_*x*_,*T*_*y*_). Based on this, we define tree *T* to be a ***join*** on an ordered pair (*T*_*x*_,*T*_*y*_) of (*k*−1)-leaf ASTs such that *T*_*x*_ and *T*_*y*_ share a common prefix, if:

(1) $$\begin{array}{l} \text{T is in canonical form, has}T_{x}\text{as its prefix, and}\\ \text{has}\;T_{y}\text{as its subtree.} \end{array}$$

Our scheme exploits condition (1) heavily. Consider equivalence classes *E* and *F*, where *E* is the parent of *F* and *E* consists of (*k*−1)-leaf ASTs. We claim that any *k*-leaf tree *T*∈*F* is the result of joining two (*k*−1)-leaf trees in *E*. Specifically, *T* is the result of joining a unique ordered pair of trees (*T*_*x*_,*T*_*y*_) in *E* such that condition (1) is satisfied. Observe that for any ordered pair (*T*_*x*_,*T*_*y*_) satisfying condition (1) with respect to *T*, *T*_*x*_ is the core tree of *F* and belongs to *E*. Further, *T*_*x*_ and *T*_*y*_ share a common prefix; thus, *T*_*y*_ also belongs to *E*. The claim follows.

While every tree in *F* can be obtained by joining a unique ordered pair (*T*_*x*_,*T*_*y*_) of trees in *E*, there may be multiple *T*s satisfying condition (1) with respect to ordered pair (*T*_*x*_,*T*_*y*_). The way in which (*T*_*x*_,*T*_*y*_) join to produce *T* depends on the topology of the subtree displayed by an input tree over the leaf set ${ℒ}_{T_{x}}\cup {ℒ}_{T_{y}}$. We next describe the four possible ways in which an ordered pair (*T*_*x*_,*T*_*y*_) can join as per condition (1). In the subsequent discussion, let *x* and *y* denote the rightmost leaf of *T*_*x*_ and *T*_*y*_ respectively, and, *p*_*x*_ and *p*_*y*_ denote the parents of *x* and *y* respectively. Recall that *E*^*c*^ represents the core tree of equivalence class *E*. Note that *E*^*c*^ is also the common prefix of *T*_*x*_ and *T*_*y*_. Let *r* denote the rightmost leaf of *E*^*c*^. For an internal node *u*, let numChild(*u*) denote its number of children. The ***rightmost path*** of a tree is the path from the root to its rightmost leaf. There are three possibilities for relative values of ${\text{depth}}^{T_{y}}(p_{y})$ and ${\text{depth}}^{T_{x}}(p_{x})$ giving rise to different types of joins.

If ${\text{depth}}^{T_{y}}(p_{y})={\text{depth}}^{T_{x}}(p_{x})$, the following three types of joins are possible. 

**Figure 7:**
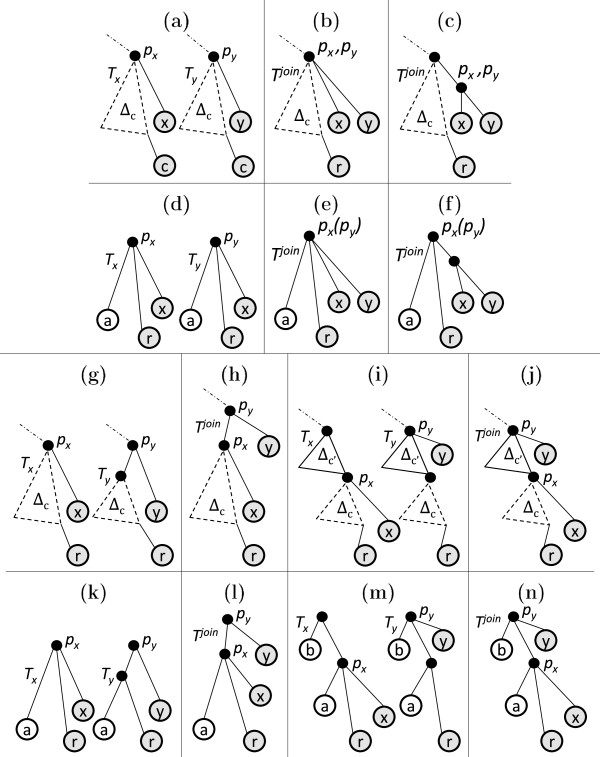
**Different types of pairwise join.** A dotted triangle represents part of the tree that may be empty, while a solid triangle represents a non-empty part of the tree. *Δ* reflects topologies of the heaviest subtrees. ‘*c*’ denotes the rightmost leaf of the common core tree. **(a)***T*_*x*_ and *T*_*y*_ in type-1 and 2 joins. **(b)** Result of type-1 join. **(c)** Result of type-2 join. **(d)** Sample inputs *T*_*x*_ and *T*_*y*_ in type-1 and 2 joins. **(e)** Result of type-1 join on sample inputs. **(f)** Result of type-2 join on sample inputs. **(g)***T*_*x*_ and *T*_*y*_ in type-3 join. **(h)** Result of type 3 join. **(i)***T*_*x*_ and *T*_*y*_ in type-4 join. **(j)** Result of type-4 join. **(k)** Sample inputs *T*_*x*_ and *T*_*y*_ in type-3 join. **(l)** Result of type 3 join on sample inputs. **(m)** Sample inputs *T*_*x*_ and *T*_*y*_ in type-4 join. **(n)** Result of type-4 join on sample inputs.

 Type 1: Figure 7(a) shows the participating trees. Leaves *x* and *y* are attached at the same depth on the rightmost path of *E*^*c*^, i.e., ${\text{depth}}^{T_{y}}(p_{y})={\text{depth}}^{T_{x}}(p_{x})$. Figure 7(b) shows the resulting join. Here, *x* and *y* are attached as siblings to the same parent node in the joined tree. Thus, for the resulting joined tree to be canonical, we must have *ψ*(*x*)<*ψ*(*y*) (recall that we assume that the labels are distinct numbers). Further, *x* and *y* are attached at the same depth in the joined tree as in *T*_*x*_ and *T*_*y*_, respectively.

*Example:* Figure 7(d) shows the input trees and Figure 7(e) shows the corresponding joined tree.

 Type 2: The input trees have the same structure as in a type 1 join (Figure 7(a)); however, in the joined tree *x* and *y* are attached to the same parent at one level deeper than their respective depths in the participating trees. See Figure 7(c).

*Example:* Figure 7(d) shows the input trees and Figure 7(f) shows the corresponding joined tree.

 Type 3: Figure 7(g) shows the participating trees. Note that the participating trees are a special case of type 1 and 2 join; i.e., ${\text{depth}}^{T_{y}}(p_{y})={\text{depth}}^{T_{x}}(p_{x})$ holds here as well. However, in the resulting join *p*_*y*_ becomes the parent of *p*_*x*_ as shown in Figure 7(h). For this to be possible, we must have numChild(*p*_*y*_)=2 in *T*_*y*_.

*Example:* Figure 7(k) shows the input trees and Figure 7(l) shows the corresponding joined tree. If ${\text{depth}}^{T_{y}}(p_{y})<{\text{depth}}^{T_{x}}(p_{x})$, the following join type arises.

 Type 4: Figure 7(i) shows the participating trees. Here ${\text{depth}}^{T_{y}}(p_{y})<{\text{depth}}^{T_{x}}(p_{x})$; i.e., on the rightmost path of *E*^*c*^, leaf *y* is attached at a lesser depth than leaf *x*. As a result there is only one way to join *T*_*x*_ and *T*_*y*_ so as to satisfy condition (1). See Figure 7(j). Here, *p*_*y*_ becomes an ancestor of *p*_*x*_ in the joined tree.

*Example:* Figure 7(m) shows the input trees and Figure 7(n) shows the corresponding joined tree.

Finally, note that if ${\text{depth}}^{T_{y}}(p_{y})>{\text{depth}}^{T_{x}}(p_{x})$, no joins are possible: *T*_*x*_ and *T*_*y*_ cannot be joined while satisfying condition (1), because *T*_*x*_ cannot be the prefix of the joined tree. ASTs from such joins are enumerated when considering the ordered pair (*T*_*y*_,*T*_*x*_).

The above scheme leads to a natural formulation for generating all members of children of *E*. For every ordered pair (*T*_*x*_,*T*_*y*_)∈*E* such that the pair joins only in one way in all the trees in the input collection, add the joined tree to $E_{T_{x}}$. The ordering indicates that the joined tree has the first tree of the ordered pair as its prefix.

### Support estimation

An AST is enumerated by combining two smaller ASTs. However, an AST can arise out of their combination only if the two ASTs exhibit a common type of join (topology) in all the input trees. Determining this involves identifying the types of joins the smaller ASTs exhibit across the input trees, and if a particular join is supported by all the input trees. For this we deploy a one-time least common ancestor based preprocessing step, after which the join type in each input tree can be identified in constant time.

Consider an ordered pair of trees (*T*_*x*_,*T*_*y*_) in an equivalence class *E* and let ${ℒ}^{\cup }={ℒ}_{T_{x}}\cup {ℒ}_{T_{y}}$. For an input tree *T*, we say the join induced by (*T*_*x*_,*T*_*y*_) in *T* is of type *A* if $T{|}_{{ℒ}^{\cup }}$ in canonical form corresponds to a type *A* join with respect to ordered pair (*T*_*x*_,*T*_*y*_). Let *T*^*j* *o* *i* *n*^ denote $T{|}_{{ℒ}^{\cup }}$ in canonical form. This step classifies *T*^*j* *o* *i* *n*^ as one of the four join types. If a particular join is supported by all the input trees (i.e. *f*=1), the corresponding joined tree is an AST. A natural way to classify the join type could be to restrict *T* to ${ℒ}^{\cup }$, canonicalize the restriction and identify the canonicalized tree as one of the four join types. This can be done in time linear in the size of *T* using the algorithm given in reference [35]. Theorem 1, stated next, improves on this by giving a least common ancestor (LCA) based scheme that in constant time identifies *T*^*j* *o* *i* *n*^ as a result of one of the four join types. The LCA values are computed as a preprocessing step. The meaning of the symbols *x*,*y*,*p*_*x*_and *p*_*y*_ is the same as in Section “Pairwise join” on page 5. Let *r* denote the rightmost leaf of core tree of *E*. Superscripts indicate the reference tree.

#### 

**Theorem 1. ***The following holds:*

1. *T*^*j* *o* *i* *n*^*is a result of a type-1 join on ordered pair*(*T*_*x*_,*T*_*y*_) *if and only if*

(a) *depth*(*LCA*^*T*^(*r*,*x*))=*depth*(*LCA*^*T*^(*r*,*y*)),

(b) *depth*(*LCA*^*T*^(*r*,*x*))=*depth*(*LCA*^*T*^(*x*,*y*)) and

(c) *ψ*(*x*)<*ψ*(*y*).

2. *T*^*j* *o* *i* *n*^*is a result of a type-2 join on ordered pair* (*T*_*x*_,*T*_*y*_) *if and only if*

(a) *depth*(*LCA*^*T*^(*r*,*x*))=*depth*(*LCA*^*T*^(*r*,*y*)),

(b) *depth*(*LCA*^*T*^(*r*,*x*))<*depth*(*LCA*^*T*^(*x*,*y*)) and

(c) *ψ*(*x*)<*ψ*(*y*).

3. *T*^*j* *o* *i* *n*^*is a result of a type-3 join on ordered pair* (*T*_*x*_,*T*_*y*_) *if and only if*

(a) ${\text{depth}}^{T_{x}}(p_{x})={\text{depth}}^{T_{y}}(p_{y})$ and

(b) *depth*(*LCA*^*T*^(*r*,*x*))>*depth*(*LCA*^*T*^(*r*,*y*)).

4. *T*^*j* *o* *i* *n*^*is a result of a type-4 join on ordered pair* (*T*_*x*_,*T*_*y*_) *if and only if*${\text{depth}}^{T_{x}}(p_{x})>{\text{depth}}^{T_{y}}(p_{y})$.

#### 

*Proof*. Let us consider each case separately. 

1. Clearly if *T*^*j* *o* *i* *n*^ is a result of a type-1 join, it satisfies 11a-11c. To prove the *only if* part, let 11a-11c be satisfied. Since *T*_*x*_ and *T*_*y*_ are obtained by attaching *x* and *y* respectively to the rightmost path of *E*^*c*^, and each is a subtree of *T*, 11a implies that depth(*p*_*y*_)=depth(*p*_*x*_). Thus, *T*^*j* *o* *i* *n*^ is a result of either a type 1, 2 or 3 join. A type-3 join requires *p*_*y*_ to be the parent of *p*_*x*_ in *T*^*j* *o* *i* *n*^, which is ruled out by 11a. Further, 11b and 11c imply that the join must be of type 1.

2. The proof is similar to that of part 1. Again, *T*^*j* *o* *i* *n*^ can be a result of either a type-1 or 2 join. Conditions 22b and 22c imply that the join must be of type 2.

3. Condition 33a implies that *T*^*j* *o* *i* *n*^ must be a result of a type-1, 2 or 3 join. Condition 33b rules out joins of type 1 and 2. Thus the join must be of type 3.

4. Follows from the definition of a type-4 join.

Cases 1– 4 are mutually exclusive and each can be evaluated in constant time as follows.

We first preprocess the input trees to answer each LCA-based query in Theorem 1 in constant time (see Section “Complexity analysis” on page 13 for more details). Using this, instead of identifying the join type in all the trees in the input collection individually, we do it in constant time across all the input trees at once. That is, in the case of ASTs, for any given ordered pair (*T*_*x*_,*T*_*y*_) we answer in constant time if a join of the pair results in an AST.

### Containing the combinatorial explosion

Although, in principle, we could enumerate all MXSTs by traversing the complete enumeration tree of ASTs, the sheer number of ASTs makes this approach, used by itself, impractical. To mitigate the impact of combinatorial explosion, we use a heuristic that, given a node in the enumeration tree, determines, without traversing the subtree below the node, whether any of its leaf descendants contains a MXST. If none of them does, we prune the branch at the node.

#### Pruning heuristic

Let *X* and *Y* be two equivalence classes. We say that *X* ***prunes the branch of the enumeration tree at*** *Y*, or simply that *X* ***prunes*** *Y*, if *X* and *Y* are of a common descent, and for every descendant *A* of *Y* (including *Y*), there exists at least one descendant *B* of *X* (which can be *X* itself) such that *B*^*c*^ displays *A*^*c*^. If *X* prunes *Y*, none of the leaf descendants of *Y* can be an MXST. Thus, the branch at *Y* need not be enumerated. For example, in Figure 6, node *A* prunes node *G* because *G* has 3 descendants —itself, *H* and *I*—, whose respective core trees are displayed by the respective core trees of nodes *B*, *C* and *D*, which are descendants of *A*. If this information is known when *G* is first visited, the branch at *G* can be pruned. Similarly, *A* also prunes *J* because the respective core trees of nodes *J*, *K* and *L* are displayed by the respective core trees of nodes *B*, *C* and *D*. Further, among the descendants of *A*, nodes *E* and *F* are respectively pruned by nodes *C* and *D*.

For the next set of results, let *E* denote an equivalence class with *r* as the rightmost leaf of its core tree. Let *X*,*Y*,*Z* be children of *E* in the enumeration tree. Let *x*,*y*,*z* be the rightmost leaves of *X*^*c*^,*Y*^*c*^,*Z*^*c*^ respectively. Clearly {*X*^*c*^,*Y*^*c*^,*Z*^*c*^}∈*E*. We say [ *i*,*j*,*k*] is an ***agreement triplet*** if all the input trees display the same topology over the leaf set {*i*,*j*,*k*}. For an ordered pair of trees (*A*,*B*) in an equivalence class, having *a* and *b* as their respective rightmost leaves, we say tree *T*_*a* *b*_ ***exists*** if (*A*,*B*) join as *T*_*a* *b*_ across all the input trees. Let *I*, *J* and *K* be three trees belonging to a common equivalence class; let *i*, *j* and *k* be their respective rightmost leaves. Suppose that *T*_*i* *j*_ and *T*_*i* *k*_ exist. If the ordered pair (*T*_*i* *j*_,*T*_*i* *k*_) exhibits a common join across all the input trees, we denote the join as *T*_*i*−*j* *k*_. Theorem 2 characterizes pruning among “siblings” and “first cousins” in the enumeration tree.

##### Theorem 2

1. *X* prunes *Y* if either of the following holds: 

(a) *T*_*x* *y*_ exists and is not of join type 2.

(b) *T*_*x* *y*_ exists as a join of type 2 and for every child *Z* of *E* such that *T*_*y* *z*_ exists, [*x*,*y*,*z*] is an agreement triplet.

2. If *T*_*x* *y*_ and *T*_*y* *z*_ exist, and *T*_*x* *y*_ is of join type 2, then $E_{T_{\text{xy}}}$ prunes $E_{T_{\text{yz}}}$ if *T*_*y* *z*_ is not of join type 2.

Part 1 of Theorem 2 deals with pruning among siblings; part 2 deals with pruning among first cousins. The proof of Theorem 2 relies on Lemmas 1, 2, 3 and 4, which we present next.

##### 

**Lemma 1. ***Suppose that T*_*x* *y*_ and *T*_*y* *z*_*exist. Then, the following holds:*

*1. If T*_*x* *y*_*is not a result of a type-2 join:*

*(a) T*_*x* *z*_*exists and is not a result of a type-2 join.*

*(b) There exists an AST T on leaf set*${ℒ}_{E^{c}}\cup \{x,y,z\}$*and*$E_{T}$*is a descendant of X*.

*2. If both T*_*x* *y*_ and *T*_*y* *z*_*are results of join type 2, and* [*x*,*y*,*z*] *is an agreement triplet:*

(a) *T*_*x* *z*_*exists.*

*(b) There exists an AST T on leaf set*${ℒ}_{E^{c}}\cup \{x,y,z\}$*and*$E_{T}$*is a descendant of X*.

*3. If T*_*x* *y*_*is a result of a type-2 join and T*_*y* *z*_*is not a result of a type-2 join:*

*(a) T*_*x* *z*_*exists and is not a result of a type-2 join.*

*(b) There exists an AST T on leaf set*${ℒ}_{E^{c}}\cup \{x,y,z\}$*and*$E_{T}$*is a descendant of*$E_{T_{\text{xy}}}$, i.e., *T*_*x* *y*−*z*_ exists.

Lemma 1 gives conditions under which for a given child $E_{T_{\text{yx}}}$ of *Y*, *X* has a descendant (specifically a grandchild) whose core tree displays *T*_*y* *x*_. Intuitively, this is an intermediate step in proving conditions under which *X* either prunes *Y* or $E_{T_{\text{yx}}}$. The specific results of Lemma 1 help in cascading the effect to further descendants of *Y* or $E_{T_{\text{yx}}}$. Each *T*_*x* *y*_ and *T*_*y* *z*_ can be a result of one of the four types of join. Thus, considering *T*_*x* *y*_ and *T*_*y* *z*_ together, there are 16 possibilities. Out of these, the case where each *T*_*x* *y*_ and *T*_*y* *z*_ is result of a type-2 join is the only case where there can be multiple topologies that display both *T*_*x* *y*_ and *T*_*y* *z*_. That is why type-2 joins have a special significance in Lemma 1. The remaining 15 cases guarantee the existence of a single topology that displays both *T*_*x* *y*_ and *T*_*y* *z*_.

##### 

**Lemma 2. ***If T*_*x* *y*_*and T*_*x* *z*_*exist, and* [ *x*,*y*,*z*] *is an agreement triplet, either T*_*x*−*y* *z*_ or *T*_*x*−*z* *y*_ exists.

Lemma 2 states that for any two children of *X* with *y* and *z* as the rightmost leaves of their respective core trees, the existence of agreement triplet [ *x*,*y*,*z*] is a sufficient condition for the children to join in one of the two possible ways. That is, there exists only one topology that displays both the children; further, the topology must be a descendant of *X*.

##### 

**Lemma 3. ***Suppose A and B are two ASTs with a and b as their respective rightmost leaves such that*${ℒ}_{A}\subset {ℒ}_{B}$. *Then,*$E_{B}$*is a descendant of*$E_{A}$*if and only if for every*$\ell \in \{{ℒ}_{B}\setminus {ℒ}_{A}\}$, *T*_*a* *ℓ*_*exists.*

##### 

**Lemma 4. ***Suppose T*_*x* *y*_*exists as a result of a type-2 join and D is descendant of Y*. *Then, for any*$\left\{a,b\}\in \{{ℒ}_{D^{c}}\setminus {ℒ}_{Y^{c}}\right\}$, [ *x*,*a*,*b*] *is an agreement triplet if* [ *x*,*y*,*a*] *and* [*x*,*y*,*b*] *are agreement triplets.*

Lemma 4 deals with the special case of type-2 joins for *T*_*x* *y*_. Intuitively, it states that for any two children of *Y* with *a* and *b* as the rightmost leaves of their respective core trees, agreement triplets [ *x*,*y*,*a*] and [ *x*,*y*,*b*] together form a sufficient condition for the existence of a descendant of *X* whose core tree displays the core trees of both children of *Y*. This is a stepping stone in proving that for every descendant *D* of *Y*, there exists a descendant of *X* whose core tree displays the core tree of *D*, thus, *X* prunes *Y*.

The proofs of the above Lemmas are given in Appendix: Proofs. We present next the proof of Theorem 2. In the proof, and in the rest of the paper, we represent trees in parenthesized Newick format (http://evolution.genetics.washington.edu/phylip/newicktree.html). E.g., (*a*,(*b*,*c*)) represents the tree with leaf set {*a*,*b*,*c*} where the LCA of *b* and *c* is a proper descendant of the LCA of *a*, *b* and *c*, and (*a*,*b*,*c*) represents unresolved (star) tree on {*a*,*b*,*c*}.

##### **Proof of Theorem 2**

1. (a) Consider any descendant *S* of *Y*. Consider any $\left\{a,b\}\in \{{ℒ}_{S^{c}}\setminus {ℒ}_{Y^{c}}\right\}$. Since *S* is a descendant of *Y*, *T*_*y* *a*_∈*Y* and *T*_*y* *b*_∈*Y* exist as per Lemma 3. Further, since *T*_*x* *y*_ is not of join type 2, as per Lemma 1.1: (i) *T*_*x* *a*_∈*X* and *T*_*x* *b*_∈*X* exist, and each is also not a result of a type 2-join, and (ii) there exists ASTs on leaf sets ${ℒ}_{E^{c}}\cup \{x,y,a\}$ and ${ℒ}_{E^{c}}\cup \{x,y,b\}$, thus, [ *x*,*y*,*a*] and [ *x*,*y*,*b*] are agreement triplets. Let *A* and *B* denote the children of *E* with *a* and *b* as their respective rightmost leaves. Since AST *S* exists, there exists an AST on leaf set ${ℒ}_{E^{c}}\cup \{a,b\}$, i.e., either *T*_*a* *b*_∈*A* or *T*_*b* *a*_∈*B* exists. Without loss of generality, let *T*_*a* *b*_ exist. Since, *T*_*x* *a*_ exists and is not a result of a type-2 join, and *T*_*a* *b*_ exists, as per Lemma 1.1, there exists an AST on leaf set ${ℒ}_{E^{c}}\cup \{x,a,b\}$. Thus: 

• for every leaf $\ell \in \{{ℒ}_{S^{c}}\setminus {ℒ}_{X^{c}}\}$, *T*_*x* *ℓ*_ exists,

• thus, for every pair of leaves {*x*_1_,*x*_2_} in

• *X*^*c*^, [ *x*_1_,*x*_2_,*ℓ*] is an agreement triplet.

• for every pair of leaves

• $\left\{a,b\}\in \{{ℒ}_{S^{c}}\setminus {ℒ}_{X^{c}}\right\}$ , there exists an AST

• on leaf set ${ℒ}_{E^{c}}\cup \{x,a,b\}$, thus, for every

• leaf *ℓ* in *X*^*c*^, [ *ℓ*,*a*,*b*] is an agreement triplet.

Thus, there exists an AST *T* on leaf set ${ℒ}_{S^{c}}\cup {ℒ}_{X^{c}}$. Clearly *T* displays *S*^*c*^. Further, for every $\ell \in \{{ℒ}_{S^{c}}\setminus {ℒ}_{X^{c}}\}$, *T*_*x* *ℓ*_ exists. Thus, by Lemma 3, *T* is descendant of *X*. Hence, *X* prunes *Y*, as claimed.

(b) Consider any descendant *S* of *Y*. Consider any $\left\{a,b\}\in \{{ℒ}_{S^{c}}\setminus {ℒ}_{Y^{c}}\right\}$. Since, *S* is a descendant of *Y*, by Lemma 3, *T*_*y* *a*_ and *T*_*y* *b*_ exists. Thus, as per the *if* condition of the claim to be proved, [ *x*,*y*,*a*] and [ *x*,*y*,*b*] are agreement triplets. Since *T*_*x* *y*_ exists as a result of type-2 join and, [ *x*,*y*,*a*] and [ *x*,*y*,*b*] are agreement triplets, by Lemma 4, [ *x*,*a*,*b*] is an agreement triplet, and, by Lemma 1 (part 2 and 3), *T*_*x* *a*_ and *T*_*x* *b*_ exist. Thus, as per Lemma 2, there exists an AST on leaf set ${ℒ}_{E^{c}}\cup \{x,a,b\}$. Thus: 

• for every leaf $\ell \in \{{ℒ}_{S^{c}}\setminus {ℒ}_{X^{c}}\}$, *T*_*x* *ℓ*_ exists,

• thus, for every pair of leaves {*x*_1_,*x*_2_} in

• *X*^*c*^, [ *x*_1_,*x*_2_,*ℓ*] is an agreement triplet.

• for every pair of leaves

• $\left\{a,b\}\in \{{ℒ}_{S^{c}}\setminus {ℒ}_{X^{c}}\right\}$ and for every leaf *ℓ* in *X*^*c*^, there exists and AST on leaf set ${ℒ}_{E^{c}}\cup \{x,a,b\}$, thus, [*ℓ*,*a*,*b*] is an agreement triplet.

Thus, there exists an AST *T* on leaf set ${ℒ}_{S^{c}}\cup {ℒ}_{X^{c}}$. Clearly *T* displays *S*^*c*^. Further, for every $\ell \in \{{ℒ}_{S^{c}}\setminus {ℒ}_{X^{c}}\}$, *T*_*x* *ℓ*_ exists, thus, by Lemma 3, *T* is descendant of *X*. Hence, *X* prunes *Y*, as claimed.

2. Since *T*_*y* *z*_ is not a result of a type-2 join, by Lemma 1.3, *T*_*x* *z*_ exists and is not a result of a type-2 join, and *T*_*x* *y*−*z*_ exists. Consider any descendant *S* of $E_{T_{\text{yz}}}$. Consider any $\ell \in \{{ℒ}_{S^{c}}\setminus {ℒ}_{T_{\text{yz}}}\}$. Since *S* is a descendant of *Y*, by Lemma 3, *T*_*y* *ℓ*_ exists. We show that *T*_*y* *ℓ*_ is not a result of a type-2 join by contradiction. Let *T*_*y* *ℓ*_ be a result of a type-2 join with triplet [*r*,*y*,*ℓ*] of type (*r*,(*y*,*ℓ*)). Since *S* is a descendant of $E_{T_{\text{yz}}}$, by Lemma 3, the join $T_{y-\mathrm{z\ell}}\in E_{T_{\text{yz}}}$ on ordered pair (*T*_*y* *z*_,*T*_*y* *ℓ*_) exists. Let *T*_*y* *z*_ be of type 1 join with triplet [ *r*,*y*,*z*] of type (*r*,*y*,*z*). Since *T*_*y*−*z* *ℓ*_ displays both (*r*,(*y*,*ℓ*)) and (*r*,*y*,*z*), it must also display (*r*,(*y*,*ℓ*),*z*). However, (*r*,(*y*,*ℓ*),*z*) cannot exist in *T*_*y*−*z* *ℓ*_ because *ℓ* and *z* cannot be the last and second-to-last leaves respectively in the IDFT. Similarly, for *T*_*y* *z*_ of join type 3 or type 4, we can show that *ℓ* cannot be the rightmost leaf in *T*_*y*−*z* *ℓ*_. Thus, *T*_*y* *ℓ*_ is not of join type 2. Thus, by Lemma 1.3, *T*_*x* *ℓ*_ exists and is not a result of type 2 join, and *T*_*x* *y*−*ℓ*_ exists. Consider any $\left\{a,b\}\in \{{ℒ}_{S^{c}}\setminus {ℒ}_{T_{\text{xy}}}\right\}$. Thus, both *T*_*x* *a*_ and *T*_*x* *b*_ exist and each is not a result of join type 2. Let *A* and *B* denote the ASTs in *E* with *a* and *b* as their rightmost leaves. Without loss of generality, let the AST on leaf set ${ℒ}_{E^{c}}\cup \{a,b\}$ be a result of a join on the order pair (*A*^*c*^,*B*^*c*^), i.e., *T*_*a* *b*_ exists. Thus, by Lemma 1.1, there exists an AST on leaf set ${ℒ}_{E^{c}}\cup \{x,a,b\}$. Thus: 

• for every $\ell \in \{{ℒ}_{S^{c}}\setminus {ℒ}_{T_{\text{xy}}}\}$, *T*_*x* *y*−*ℓ*_ exists, thus, for every pair of leaves {*x*_1_,*x*_2_} in *T*_*x* *y*_, [ *x*_1_,*x*_2_,*ℓ*] is an agreement triplet.

• for every $\left\{a,b\}\in \{{ℒ}_{S^{c}}\setminus {ℒ}_{T_{\text{xy}}}\right\}$ and for every leaf *ℓ* in *T*_*x* *y*_, there exists an AST on leaf set ${ℒ}_{E^{c}}\cup \{x,a,b\}$, thus, [ *ℓ*,*a*,*b*] is an agreement triplet.

Thus, there exists an AST *T* on leaf set ${ℒ}_{S^{c}}\cup {ℒ}_{T_{\text{xy}}}$. Clearly *T* displays *S*^*c*^. Further, since for every $\ell \in \{{ℒ}_{S^{c}}\setminus {ℒ}_{T_{\text{xy}}}\}$, *T*_*x* *y*−*ℓ*_ exists, by Lemma 3, *T* is descendant of $E_{T_{\text{xy}}}$. Hence, $E_{T_{\text{xy}}}$ prunes $E_{T_{\text{xz}}}$, as claimed.

□

##### Pruner-list

Note that conditions in part (1a) and part (2) of Theorem 2 can be evaluated in constant time while testing the condition in part (1b) of Theorem 2 takes time linear in the size of the tree being pruned. Neither of these require enumerating the pruned branch at *Y*. However, there are cases when *Y* or a descendant of *Y* is pruned by *X* but it cannot be identified using Theorem 2. In this case, the branch at *Y* must be enumerated and potential pruning by *X* verified. For this, we maintain a “pruner-list” for every child of *Y*. We explain this idea next.

Let *X*,*Y*,*Z* be children of an equivalence class *E* in the enumeration tree. Let *x*,*y*,*z* be the rightmost leaves of *X*^*c*^,*Y*^*c*^,*Z*^*c*^ respectively. Let joins *T*_*x* *y*_,*T*_*y* *z*_ exist such that none of the cases of Theorem 2 hold. Then the *pruner-list* of $E_{T_{\text{yz}}}$:

(2) contains x if [x,y,z] is an agreement triplet.

(3) $$\begin{array}{l} \text{inherits members from the intersection of the}\\ \text{pruner lists of}\;E_{T_{y}}\;\text{and}\;E_{T_{z}}. \end{array}$$

Now, using arguments similar to the proof of Theorem 2, we can show that:

###### Theorem 3

*Y* is pruned by an equivalence class *A* with *a* as the rightmost leaf of *A*^*c*^ if either of the following holds: 

1. *Y* has no children and has *a* in its pruner-list.

2. All children of *Y* have *a* in their pruner-list.

#### MFSTMINER

Algorithm 1 is a high-level description of MFSTMINER for the special case of enumerating all MXSTs for a collection *C* of input trees. MFSTMINER first invokes ENUMERATEAST_TRIPLETS (whose details are omitted) to enumerate all AST triplets and to partition them into equivalence classes. The set of all such classes is denoted by *EC*_3_. Note that each equivalence class in *EC*_3_ is a child of the root of the enumeration tree. After this, MFSTMINER invokes subroutine ENUMERATENODE, explained next, to enumerate the elements of the branch at each equivalence class in *EC*_3_. 

Algorithm 2 shows the details of ENUMERATENODE, which accepts an equivalence class *E* as input and enumerates the branch at *E* in the enumeration tree. In the pseudocode, *T*_*x*_ denotes an AST that has *x* as its rightmost leaf. Lines 3-5 perform pair-wise joining among members of an equivalence class in the enumeration tree. Comments in braces indicate where the algorithm performs assignment to pruner-lists, as per conditions (2) and (3), and pruning, according to the different cases of Theorems 2 and 3. In line 12, the core tree of an empty equivalence class — representing a leaf node — is produced as output if it is found to be not pruned. 

In line 17, ENUMERATENODE calls itself recursively to enumerate the children of a non-empty equivalence class $E_{T_{x}}$, provided $E_{T_{x}}$ is found to be not pruned.

#### The general case of enumerating MFSTs

We now explain how MFSTMINER handles the general case of mining MFSTs. The main difference between mining for MXSTs and mining for MFSTs is that the former is (as we have seen) based on enumerating ASTs, while the latter is based on enumerating FSTs. While an ASTs must be supported by all the input trees (i.e., *f*=1), an FSTs need only be supported by some fraction $f\in \left(\frac{1}{2},1\right)$ of the input trees. This difference affects neither the enumeration tree nor the pairwise join, but it does affect support estimation and the pruning strategy. We discuss these steps next.

##### Support estimation

Given *T*_*x*_ and *T*_*y*_ in an equivalence class, a join *T*_*x* *y*_ on ordered pair (*T*_*x*_,*T*_*y*_) is an FST if it is supported by at least a fraction *f* of the input trees (i.e., if at least a fraction *f* of the input trees have *T*_*x* *y*_ as a subtree). Note that any such *T*_*x* *y*_ is supported only by those trees that support both *T*_*x*_ and *T*_*y*_ as well. Motivated by this, for each FST *T*_*x*_ we maintain a *support list*, denoted by *T*_*x*_.supList, that contains all trees in the input collection that support *T*_*x*_. To estimate if the join on (*T*_*x*_,*T*_*y*_) results in an FST, we apply Theorem 1 only on trees in *T*_*x*_.supList∩*T*_*y*_.supList. We store the support list as a bitmap [36] for efficient memory utilization and fast computation of intersection of support lists.

##### Pruning strategy

To verify whether an equivalence class *X* prunes an equivalence class *Y*, we also need to consider the support lists of *X*^*c*^ and *Y*^*c*^. We say [ *x*,*y*,*z*] is a *frequent triplet* if at least fraction *f* of the input trees display the same triplet over the leaf set {*x*,*y*,*z*}. Let [ *x*,*y*,*z*].supList denote the support list of such a frequent triplet. Based on this, we can restate Theorem 2 for the case of enumerating MFSTs as follows.

###### Theorem 4

1. *X* prunes *Y* if either 

(a) *T*_*x* *y*_ exists, *Y*^*c*^.supList⊆*X*^*c*^.supList and *T*_*x* *y*_ is not of join type 2, or

(b) *T*_*x* *y*_ exists as a type-2 join, *Y*^*c*^.supList⊆*X*^*c*^.supList and for every *Z*∈*E* such that *T*_*y* *z*_ exists, [ *x*,*y*,*z*] is a frequent triplet with *Y*^*c*^.supList⊆[ *x*,*y*,*z*].supList.

2. If *T*_*x* *y*_ and *T*_*y* *z*_, exist and *T*_*x* *y*_ is of join type 2, then $E_{T_{\text{xy}}}$ prunes $E_{T_{\text{yz}}}$ if *T*_*y* *z*_ is not of join type 2 and *T*_*y* *z*_.supList⊆*T*_*x* *y*_.supList.

##### Pruner-list

Pruning cases not identified by Theorem 4 require the use of pruner-list. In the case of MFSTs, along with leaf label the pruner-list also contains the support list of the core tree of the equivalence class that is claiming to prune. To explain further, let *T*_*x*_,*T*_*y*_,*T*_*z*_ be FSTs in an equivalence class *E* with *x*,*y*,*z* as their respective rightmost leaves, such that joins *T*_*x* *y*_ and *T*_*y* *z*_ exist, and none of the cases of Theorem 4 hold. For an equivalence class *A*, let *A*.prunerList denote its pruner-list. Then, the next set of conditions describe pruner-lists for enumerating MFSTs. 

1. $E_{T_{\text{yz}}}.\text{prunerList}$ contains the entry (*x*,*T*_*x* *y*_.supList) if *T*_*x* *y*_ is not of join type 2 and |*T*_*x* *y*_.supList∩*T*_*y* *z*_.supList|≥*f*.

2. $E_{T_{\text{yz}}}.\text{prunerList}$ contains the entry (*x*,*S*_∩_=*T*_*x* *y*_.supList∩[ *x*,*y*,*z*].supList) if *T*_*x* *y*_ is of join type 2, [ *x*,*y*,*z*] is a frequent triplet and |*S*_∩_∩*T*_*y* *z*_.supList|≥*f*.

3. For every leaf label *w* such that $(w,S_{\cap }^{y})\in E_{T_{y}}.\text{prunerList}$ and $(w,S_{\cap }^{z})\in E_{T_{z}}.\text{prunerList}$ exist, $E_{T_{\text{yz}}}.\text{prunerList}$ contains entry $\left(w,S_{\cap }=S_{\cap }^{y}\cap S_{\cap }^{z}\right)$ if |*S*_∩_∩*T*_*y* *z*_.supList|≥*f*.

Condition 1 and 2 describe addition of new labels to the pruner-list of $E_{T_{\text{yz}}}$, while condition 3 describes inheritance of labels from the intersection of the pruner-lists of $E_{T_{y}}$ and $E_{T_{z}}$. Now, the corresponding result for Theorem 3 can be stated as:

###### Theorem 5

Given an equivalence class *A* with *a* as the rightmost leaf of *A*^*c*^, 

1. $E_{T_{\text{yz}}}$ is pruned by *A* if (i) for every $T_{\text{yb}}\in E_{T_{y}}$, $E_{T_{\text{yb}}}.\text{prunerList}$ contains an entry (*a*,*S*_*b*_(*a*)), and (ii) for $S_{\cap }=\underset{T_{\text{yb}}\in E_{T_{y}}}{⋂}S_{b}(a)$, $E_{T_{\text{yz}}}.\text{supList}=S_{\cap }$.

2. $E_{T_{y}}$ is pruned by *A* if (i) $E_{T_{y}}$ is empty and has *a* in its pruner list, or (ii) every child $E_{T_{\text{yb}}}$ of $E_{T_{y}}$ is pruned by *A* as per part 1 of this Theorem.

This completes the description of MFSTMINER for the general case of mining MFSTs. The overall framework is the same as the special case of mining all MXSTs. The difference lies in the finer details of incorporating support lists in the support estimation and the pruning step. These details were discussed in this section and are easy to incorporate in Algorithms 1 and 2.

### Complexity analysis

Here we discuss the runtime complexity of the preprocessing step, the data structures used in the algorithm implementation and the memory requirements in each step of MFSTMINER algorithm. In the following discussion, let the input collection consist of *n* trees on a common leaf set  and let *f* denote the input fraction for computing *f*-frequent subtrees.

#### 

##### Preprocessing

This one-time task involves (1) computing LCA mappings for all pairs of leaves for all the input trees and (2) enumerating all frequent triplets. For each tree *T* in the input collection, and every pair {*u*,*v*} of leaves of *T*, step (1) computes LCA^*T*^(*u*,*v*) and stores it in a three-dimensional array indexed by triplet (*T*,*u*,*v*), thus, requires $O(n|ℒ{|}^{2})$ space. In our implementation, these LCA values are computed in quadratic time and space per tree by traversing the tree in a depth-first manner and computing the LCA values of the leaf-descendants at a node. Thus, for all the input trees, Step 1 takes $O(n|ℒ{|}^{2})$ time and space. In the case of MXSTs only a two-dimensional array is used (requiring $O(|ℒ{|}^{2})$ space) to store the LCA values because an LCA value is relevant only if it is the same across all input trees, i.e., *f*=1. We should point out that it is well-known that one can pre-process a tree in linear time and space to produce a data structure that can answer any LCA query on that tree in constant time [37-39]. Such algorithms are quite useful when the number of LCA queries is limited and the pre-processing dominates the total time. That is not the case in our application. Indeed, MFSTMINER queries all possible LCA values while enumerating all MFSTs on three leaves, and then does a constant number of LCA queries for every join operation thereafter. Although both our three-dimensional array and the specialized LCA data structures [37-39] offer constant-time access to LCA-values, the former’s constant factor is smaller than the latter’s, which makes a significant difference in practice.

Step 2 takes $O(n|ℒ{|}^{3})$ time and space, and uses the precomputed LCA values from step (1). In the case of MXSTs, just as storing of LCA values requires less space ($O(|ℒ{|}^{2})$), the complexity in step (2) is also reduced: $O(|ℒ{|}^{3})$ time and space.

##### Join-operation

Every join operation requires a constant number of LCA queries and depths of certain nodes (see Theorem 1). However, note that the relative depths are needed rather than absolute values. In most of the cases of Theorem 1 the relative depths can be obtained by examining the type of tree a certain triplet displays. That is, Theorem 1 can be restated as: 

1. *T*^*j* *o* *i* *n*^ is a result of a type-1 join on ordered pair (*T*_*x*_,*T*_*y*_) if and only if 

(a) the triplet on leaves {*r*,*x*,*y*} is of type (*r*,*x*,*y*) and

(b) *ψ*(*x*)<*ψ*(*y*).

2. *T*^*j* *o* *i* *n*^ is a result of a type-2 join on ordered pair (*T*_*x*_,*T*_*y*_) if and only if 

(a) the triplet on leaves {*r*,*x*,*y*} is of type (*r*,(*x*,*y*)) and

(b) *ψ*(*x*)<*ψ*(*y*).

3. *T*^*j* *o* *i* *n*^ is the result of a type-3 join on ordered pair (*T*_*x*_,*T*_*y*_) if and only if 

(a) ${\text{depth}}^{T_{x}}(p_{x})={\text{depth}}^{T_{y}}(p_{y})$ and

(b) the triplet on leaves {*r*,*x*,*y*} is of type ((*r*,*x*),*y*).

4. *T*^*j* *o* *i* *n*^ is a result of a type-4 join on ordered pair (*T*_*x*_,*T*_*y*_) if and only if 

(a) ${\text{depth}}^{T_{x}}(p_{x})>{\text{depth}}^{T_{y}}(p_{y})$ and

(b) the triplet on leaves {*r*,*x*,*y*} is of type ((*r*,*x*),*y*).

The advantage of reformulating Theorem 1 as above is that (a) for every tree in the input collection, we need not store the depth of its nodes, and (b) for every FST enumerated as a result of a join operation, we only need to store the depth of the parent of its rightmost leaf. Further, the type of tree a certain triplet displays can be easily known through a constant number of queries on precomputed LCA values.

##### Support-list and Pruner-list

In the case of MXSTs, there is no support-list and the pruner-list contains leaf labels. This takes O(|ℒ|) space per enumerated AST that is currently in memory. Note that MFSTMINER uses a depth-first strategy for traversing the enumeration tree. Thus, not all enumerated ASTs are held in memory at any given time. In the case of MFSTs, both support-list and pruner-list exist. The support-list for an FST contains the list of input trees that display the FST. This takes *O*(*n*/*w*) space per enumerated AST that is currently in memory, where *w* is the size of the machine word. The *i*^*t* *h*^ bit of a support-list is set to 1 if the corresponding FST is displayed by the *i*^*t* *h*^ tree in the input collection, and 0 otherwise. Our experiments were run on a *w*=64 bit processor, which is typically the case with any modern computer. An entry in pruner-list contains a leaf-label and a support-list. This takes O((n/w)+|ℒ|) space per enumerated AST that is currently in memory. These are worst-case estimates and in practice the memory consumption is much less because pruner-list is required only for cases not captured by Theorems 2 and 4.

The next result shows that the memory required by MFSTMINER scales polynomially with the number of input trees and the size of the common leaf set.

###### Theorem 6

MFSTMINER requires $O(n|ℒ{|}^{3})$ space in the case of enumerating MFSTs and $O(|ℒ{|}^{3})$ space when only MXSTs are being enumerated.

###### *Proof*.

The enumeration tree has depth at most |ℒ|. Enumerating an FST at this depth will require storing O(|ℒ|) ancestor equivalences classes, each of which can have at most |ℒ| FSTs. Thus, the maximum number of FSTs to be stored is $O(|ℒ{|}^{2})$. Storing each such FST requires O(|ℒ|) space for the subtree, *O*(*n*/*w*) space for the support-list and O((n/w)+|ℒ|) space for the pruner-list, where *w* is the size of the machine word. Thus, the maximum space required to store all FSTs is $O(((n/w)+|ℒ|)|ℒ{|}^{2})$. Adding to this the space required to store LCA mappings, which is $O(n|ℒ{|}^{3})$, we get the claimed figure. Similarly, it can be shown that in the case of MXSTs, MFSTMINER requires $O(|ℒ{|}^{3})$ space.

Let |F| be the number of *FSTs* (not MFSTs) the input collection displays. Then, the worst-case time complexity of MFSTMINER is O(n|F|+|ℒ||F|), which is the same as EVOMINER’s [10]. The time complexity is not polynomial in the number of *MFSTs*, because we cannot polynomially bound the number of FSTs that MFSTMINER will enumerate to verify the pruning of an equivalence class. Indeed, it could happen that *X* prunes *Y*, but that this cannot be confirmed by Theorems 2 and 4. If so, MFSTMINER must enumerate the branch at *Y* further, and there is no polynomial bound on the number of FSTs that would have to be generated to verify the pruning of *Y* by *X*.

Nonetheless, as we will see in the next section, even though MFSTMINER shares the same worst-case time complexity with EVOMINER, it can be orders or magnitudes faster than EVOMINER in practice.

## Results and discussion

To study the effectiveness of MFSTMINER, we conducted four categories of experiments: 

1. Comparison of MFSTs with MASTs.

2. Comparison of MfstMiner with EvoMiner[10] — the state-of-the-art algorithm for enumerating all phylogenetic FSTs.

3. Evaluation of the scalability of MfstMiner with respect to the number of trees, the size of the leaf set and the support value.

4. Comparison with Ramu et al.’s [23] approach that mines MFSTs having maximum leaves.

Our dataset consists of bootstrapped trees from a previous study [40] on bootstrapping methods. There are seventeen sets of trees constructed from a diverse range of sequences including rbcL genes, mammalian sequences, bacterial and archaeal sequences, ITS sequences, fungal sequences, and grasses. The number of taxa in these single-gene and multi-gene DNA sequences vary from 125-2554. The entire dataset is available at http://lcbb.epfl.ch/BS.tar.bz2. We refer to the seventeen datasets as *A*–*Q* in the increasing order of taxa in the DNA sequences from which the trees were constructed. *A* corresponds to the set of trees with 125 taxa and *Q* corresponds to the set of trees with 2554 taxa. To extract datasets with different numbers of leaves and trees, we randomly selected the required number of trees and restricted them on a random set of leaves of the required size.

The experiments were split over 4 machines: 

•Two machines running Windows 7 64-bit with processor clock-speed of 3.4 GHz, 4 cores and 8 threads.

•One machine running Windows 7 64-bit with processor clock-speed of 3.16 GHz and 2 cores.

•One machine running Linux 64-bit with processor clock-speed of 2.0 GHz, 6 cores and 12 threads.

Each experiment was averaged over 5 runs. For practical purposes, each run was allowed a maximum of 10000 seconds. Thus, any missing entry in the graphs indicate that the corresponding experiment took more than this limit. Initially, we started with all experiments on one machine and using only one core at a time — the ideal environment — to allow maximum fairness in comparing results, however, we soon realized that this would take more than 600 days of runtime. Thus, we split the experiments using maximum number of cores and threads on each machine. This did slow down things because of competing memory and disk requirements, however, the final runtimes should not be more than a factor of two of the runtimes in the ideal environment.

### 

#### 

##### MFSTs vs. MASTs

**Figure 8:**
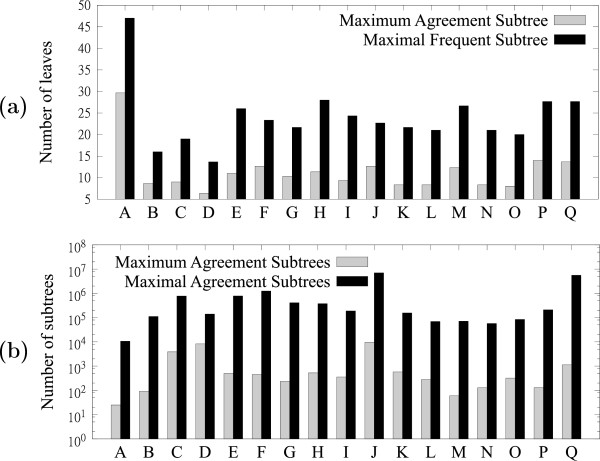
**Utility of MFSTs over MASTs.(a)** MFSTs have more leaves than MASTs; thus, they reveal common agreement over a larger set of taxa than MASTs. **(b)** MXSTs are more numerous than MASTs; thus, they reveal more agreement agreement information than MASTs.

Figure 8(a) compares the size of the MAST with the size of the largest MFST. This experiment was conducted on a set of 100 trees on 50 leaves from each of the datasets. MFSTs were enumerated for *f*=0.51. In some cases the largest MFST is more than twice as big as the corresponding MAST.

Figure 8(b) compares the number of MASTs with the number of MFSTs for *f*=1. There are significantly more MFSTs. This is notable, because any MFST that is not a MAST is also not displayed by any of the MASTs. Thus, such a MFST reveals unique agreement information among the input trees. This experiment was conducted on a set of 100 trees on 100 leaves from each of the datasets.

##### Comparison with **EVOMINER**.

**Figure 9:**
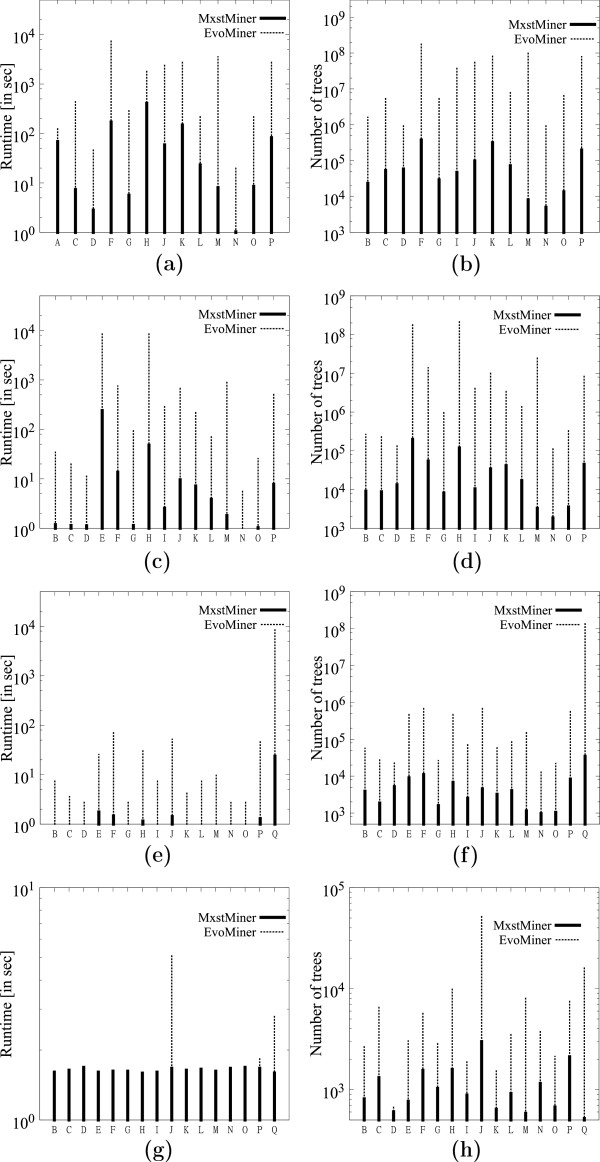
**Comparison with **EVOMINER.**(a)** Runtime comparison for *f*=.55. **(b)** Number of subtrees enumerated for *f*=.55. **(c)** Runtime comparison for *f*=.75. **(d)** Number of subtrees enumerated for *f*=.75. **(e)** Runtime comparison for *f*=.95. **(f)** Number of subtrees enumerated for *f*=.95. **(g)** Runtime comparison for *f*=1.0. **(h)** Number of subtrees enumerated for *f*=1.0.

This experiment was conducted on a set of 1000 trees on 40 leaves from each of the datasets. Figure 9(a) compares MFSTMINER with EVOMINER[10] for *f*=.55 with respect to runtime. Figure 9(b) shows the corresponding number of MFSTs and FSTs mined by MFSTMINER and EVOMINER respectively. Figures 9(c)-(d), 9(e)-(f), and 9(g)-(h) show the corresponding figures for support *f*=.75, *f*=.95 and *f*=1.0 respectively. We see that enumerating MFSTs can very often be orders of magnitude faster than enumerating all FSTs. The time difference arises due to the number of subtrees mined. The ratio of the number of subtrees mined by EVOMINER to the number of subtrees mined by MFSTMINER is maximum for support values *f*=.75 and *f*=.95, thus, MFSTMINER is fastest with respect to EVOMINER in these cases. For, *f*=1.0EVOMINER is often faster than MFSTMINER. We believe this is because (a) the runtimes are too small for a fair comparison and thus, the pre-processing time (enumerating all frequent triplets), which is same for both EVOMINER and MFSTMINER, dominates the total time, and (b) some implementation inefficiency in MFSTMINER is suspected. The missing dataset entries correspond to cases where EVOMINER took more than 10000 seconds.

##### Scalability of **MFSTMINER**

We evaluated the scalability of MFSTMINER with respect to the number of leaves (10-250), the number of trees (100-10000) and the support value (.51-1.0) on datasets having at least 250 leaves, i.e., datasets *D* (354 taxa) — *Q* (2554 taxa). Presenting results for all datasets would have been overwhelming, thus, we discuss results for datasets *D* (354 taxa), *K* (1481 taxa) and *Q* (2554 taxa) — the first, the last and a middle one from datasets *D*-*Q*.

**Figure 10:**
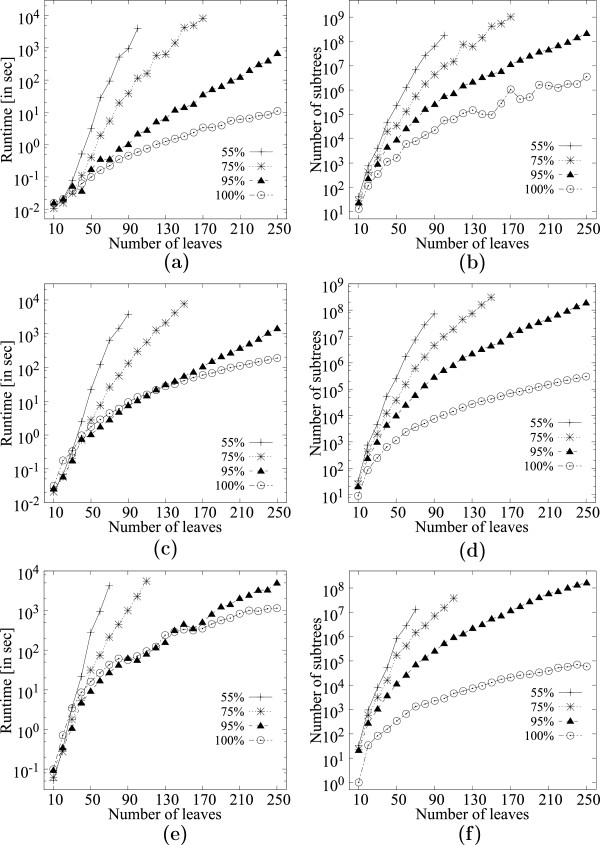
**Scalability of **MFSTMINER** on dataset D (354 taxa) while varying the number of leaves in the input trees.(a)** Runtime comparison on 200 input trees. **(b)** Number of subtrees enumerated on 200 input trees. **(c)** Runtime comparison on 1000 input trees. **(d)** Number of subtrees enumerated on 1000 input trees. **(e)** Runtime comparison on 5000 input trees. **(f)** Number of subtrees enumerated on 5000 input trees.

Figure 10(a) shows the runtime for 200 trees, with the number of leaves varying from 10-250, for support values *f*=.55, *f*=.75, *f*=.95 and *f*=1.0 on dataset *D*. Figure 10(b) shows the corresponding number of MFSTs mined. Figures 10(c)-(d) and 10(e)-(f) show the corresponding results for 1000 and 5000 trees respectively. The results show that for a given number of trees, the number of subtrees mined increases steadily with the increase in the number of leaves in the input trees, while the runtime follows closely the number of subtrees mined.

**Figure 11:**
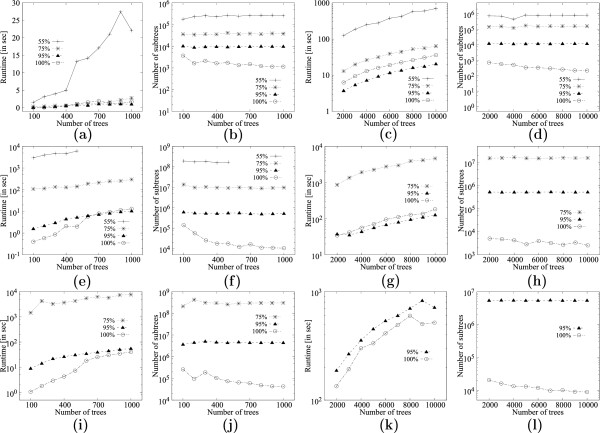
**Scalability of **MFSTMINER** on dataset *D * (354 taxa) while varying the number of input trees.(a)** Runtime comparison with 50 leaves in the input trees while varying the number of input trees from 100 to 1000. **(b)** Number of subtrees enumerated with 50 leaves in the input trees while varying the number of input trees from 100 to 1000. **(c)** Runtime comparison with 50 leaves in the input trees while varying the number of input trees from 2000 to 10000. **(d)** Number of subtrees enumerated with 50 leaves in the input trees while varying the number of input trees from 2000 to 10000. **(e)** Runtime comparison with 100 leaves in the input trees while varying the number of input trees from 100 to 1000. **(f)** Number of subtrees enumerated with 100 leaves in the input trees while varying the number of input trees from 100 to 1000. **(g)** Runtime comparison with 100 leaves in the input trees while varying the number of input trees from 2000 to 10000. **(h)** Number of subtrees enumerated with 100 leaves in the input trees while varying the number of input trees from 2000 to 10000. **(i)** Runtime comparison with 150 leaves in the input trees while varying the number of input trees from 100 to 1000. **(j)** Number of subtrees enumerated with 150 leaves in the input trees while varying the number of input trees from 100 to 1000. **(k)** Runtime comparison with 150 leaves in the input trees while varying the number of input trees from 2000 to 10000. **(l)** Number of subtrees enumerated with 150 leaves in the input trees while varying the number of input trees from 2000 to 10000.

Figure 11(a) evaluates the variation in runtime for 50 leaves on 100-1000 trees for support values *f*=.55, *f*=.75, *f*=.95 and *f*=1.0 on dataset *D*. Figure 11(b) shows the corresponding number of MFSTs mined. Figures 11(c) and 11(d) show the corresponding values while varying the number of trees from 2000-10000. Figures 11(e)-(h) and 11(i)-(l) show the corresponding results for input trees with 100 and 150 leaves respectively. The results show that for a given number of leaves, the number of subtrees very much remain the same as the number of input trees is varied, while the runtime increases steadily with increase in the number of input trees. This is expected because the support estimation takes loner with more trees.

**Figure 12:**
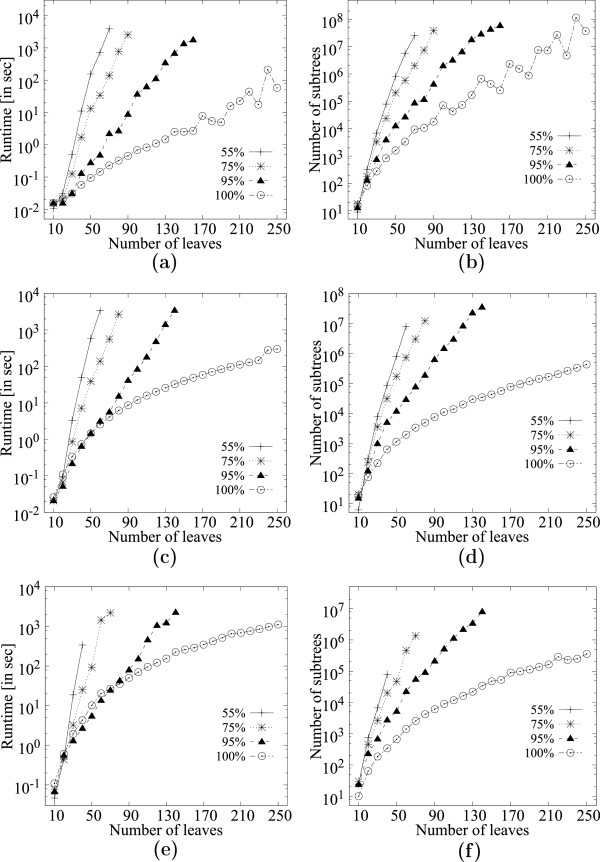
**Scalability of **MFSTMINER** on dataset*K* (1481 taxa) while varying the number of leaves in the input trees.(a)** Runtime comparison on 200 input trees. **(b)** Number of subtrees enumerated on 200 input trees. **(c)** Runtime comparison on 1000 input trees. **(d)** Number of subtrees enumerated on 1000 input trees. **(e)** Runtime comparison on 5000 input trees. **(f)** Number of subtrees enumerated on 5000 input trees.

**Figure 13:**
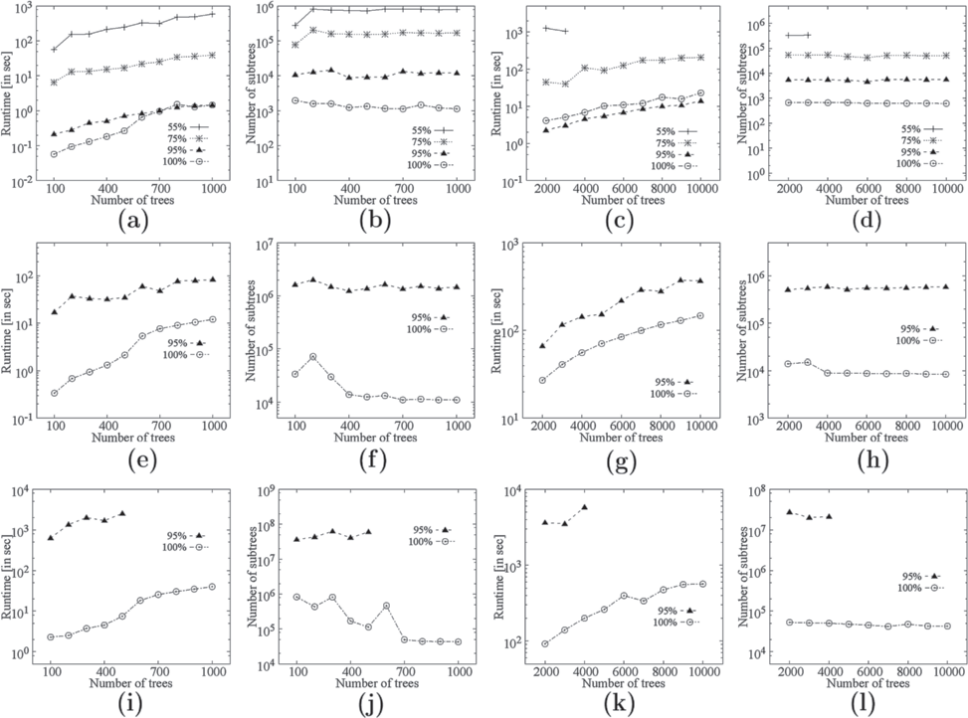
**Scalability of **MFSTMINER** on dataset*K* (1481 taxa) while varying the number of input trees.(a)** Runtime comparison with 50 leaves in the input trees while varying the number of input trees from 100 to 1000. **(b)** Number of subtrees enumerated with 50 leaves in the input trees while varying the number of input trees from 100 to 1000. **(c)** Runtime comparison with 50 leaves in the input trees while varying the number of input trees from 2000 to 10000. **(d)** Number of subtrees enumerated with 50 leaves in the input trees while varying the number of input trees from 2000 to 10000. **(e)** Runtime comparison with 100 leaves in the input trees while varying the number of input trees from 100 to 1000. **(f)** Number of subtrees enumerated with 100 leaves in the input trees while varying the number of input trees from 100 to 1000. **(g)** Runtime comparison with 100 leaves in the input trees while varying the number of input trees from 2000 to 10000. **(h)** Number of subtrees enumerated with 100 leaves in the input trees while varying the number of input trees from 2000 to 10000. **(i)** Runtime comparison with 150 leaves in the input trees while varying the number of input trees from 100 to 1000. **(j)** Number of subtrees enumerated with 150 leaves in the input trees while varying the number of input trees from 100 to 1000. **(k)** Runtime comparison with 150 leaves in the input trees while varying the number of input trees from 2000 to 10000. **(l)** Number of subtrees enumerated with 150 leaves in the input trees while varying the number of input trees from 2000 to 10000.

**Figure 14:**
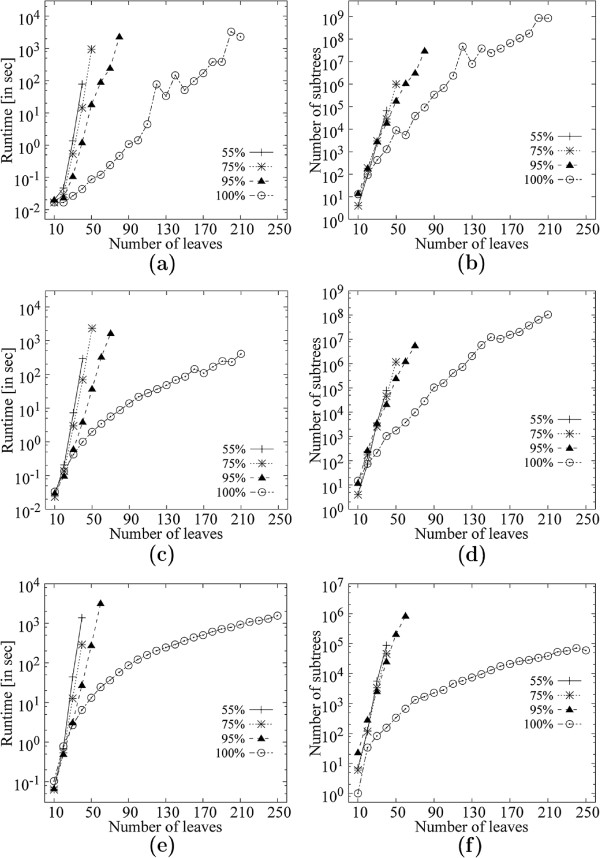
**Scalability of **MFSTMINER** on dataset*Q* (2554 taxa) while varying the number of leaves in the input trees.(a)** Runtime comparison on 200 input trees. **(b)** Number of subtrees enumerated on 200 input trees. **(c)** Runtime comparison on 1000 input trees. **(d)** Number of subtrees enumerated on 1000 input trees. **(e)** Runtime comparison on 5000 input trees. **(f)** Number of subtrees enumerated on 5000 input trees.

**Figure 15:**
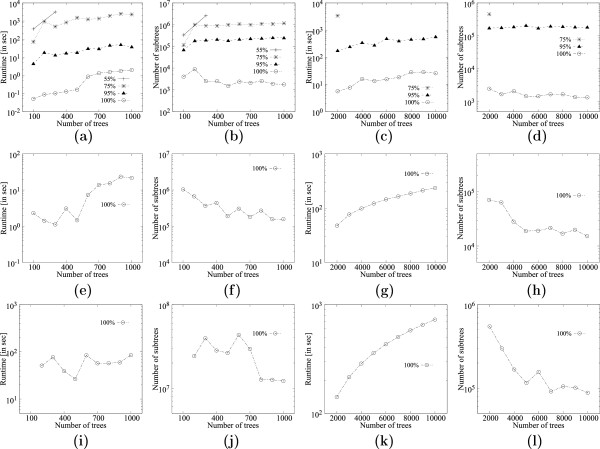
**Scalability of **MFSTMINER** on dataset *Q * (2554 taxa) while varying the number of input trees.(a)** Runtime comparison with 50 leaves in the input trees while varying the number of input trees from 100 to 1000. **(b)** Number of subtrees enumerated with 50 leaves in the input trees while varying the number of input trees from 100 to 1000. **(c)** Runtime comparison with 50 leaves in the input trees while varying the number of input trees from 2000 to 10000. **(d)** Number of subtrees enumerated with 50 leaves in the input trees while varying the number of input trees from 2000 to 10000. **(e)** Runtime comparison with 100 leaves in the input trees while varying the number of input trees from 100 to 1000. **(f)** Number of subtrees enumerated with 100 leaves in the input trees while varying the number of input trees from 100 to 1000. **(g)** Runtime comparison with 100 leaves in the input trees while varying the number of input trees from 2000 to 10000. **(h)** Number of subtrees enumerated with 100 leaves in the input trees while varying the number of input trees from 2000 to 10000. **(i)** Runtime comparison with 150 leaves in the input trees while varying the number of input trees from 100 to 1000. **(j)** Number of subtrees enumerated with 150 leaves in the input trees while varying the number of input trees from 100 to 1000. **(k)** Runtime comparison with 150 leaves in the input trees while varying the number of input trees from 2000 to 10000. **(l)** Number of subtrees enumerated with 150 leaves in the input trees while varying the number of input trees from 2000 to 10000.

Figures 12, 13, 14 and 15 show the corresponding results for datasets *K* and *Q* respectively. The trends are similar to dataset *D* except that dataset *Q* seems to produce much more MFSTs, thus, the runtimes are larger. Results from dataset *K* seem to lie somewhere in the middle of the range of results from datasets *D* and *K*.

The above results also show that MFSTMINER can handle much larger datasets than EVOMINER. Again, the missing entries are due to the 10000 second time limit. However, if time is not a constraint, as discussed before, the memory requirements of MFSTMINER is polynomial in the size of the input, thus, it can handle large datasets.

##### Comparison with Ramu et al.’s approach

We compared our approach with Ramu et al.’s [23] heuristic approach that mines MFSTs with maximum leaves. We used the original implementation shared by the authors. The current implementation mines only one MFST with maximum leaves, thus, we cannot compare the number of subtrees mined by our approach with theirs. Further, the current implementation has major portions written in Perl, a high-level interpreted programming language, and involves significant disk usage for storing intermediate data-structures, whereas, our implementation is written in C++, a much lower-level compiled programming language, and keeps all intermediate data-structures in memory. Thus, we did not compare the runtime because our implementation has much advantage with respect to the speed of execution. We compare the size of the MFST with maximum leaves mined by Ramu et al.’s [23] implementation with ours. We did this comparison on a set of 100 trees with 20 leaves from each of the datasets for support values *f*=.55 and *f*=.75. In 25 out of 34 cases, the size of the MFST with maximum leaves returned by Ramu et al.’s [23] approach was at least as good as ours. Only in 9 cases it returned an MFST with one leaf less than ours. So, if the goal is to get an MFST with most leaves, Ramu et al.’s [23] approach seems near-perfect. As mentioned in their paper [23], it also seems to be capable of handling large datasets in terms of the number of trees and the number of leaves. However, if the goal is to mine all MFSTs with maximum leaves or simply all MFSTs, then our approach serves better. This can be very useful because there can be a lot of MFSTs (either with maximum leaves or all of them), and every MFST returned by our approach conveys some unique agreement information not conveyed by any of remaining returned MFSTs.

## Conclusions

Although we have restricted our attention to enumerating MFSTs for $f\in \left(\frac{1}{2},1\right]$, we can extend MFSTMINER to enumerate all MFSTs for $f\in \left(0,\frac{1}{2}\right]$, with small modifications in the pruning strategy. Note, however, that when $f\in \left(0,\frac{1}{2}\right]$ there can potentially be different MFSTs with the same leaf set.

As a future work, we intend to do a thorough comparison of MFSTs against MASTs, in the settings where MAST is currently used [2,5,7]. Since the time to enumerate MFSTs for larger leaf sets can be prohibitive, we also intend to develop schemes to sample at random from the set of all MFSTs.

An intriguing open problem is to devise methods to find common patterns in collections of phylogenetic networks [41-44]. Although techniques from maximal subgraph mining [16,17] may prove useful here, the special characteristics of phylogenetic networks add interesting twists to the problem. We also intend to extend our work for mining frequent sub-structures in multi-labeled trees [45-48].

The current implementation of MFSTMINER, which works for up to 250 leaves and 10000 trees, is available at https://code.google.com/p/mfst-miner/.

## Appendix: Proofs

### **Proof of Lemma 1**

**Figure 16:**
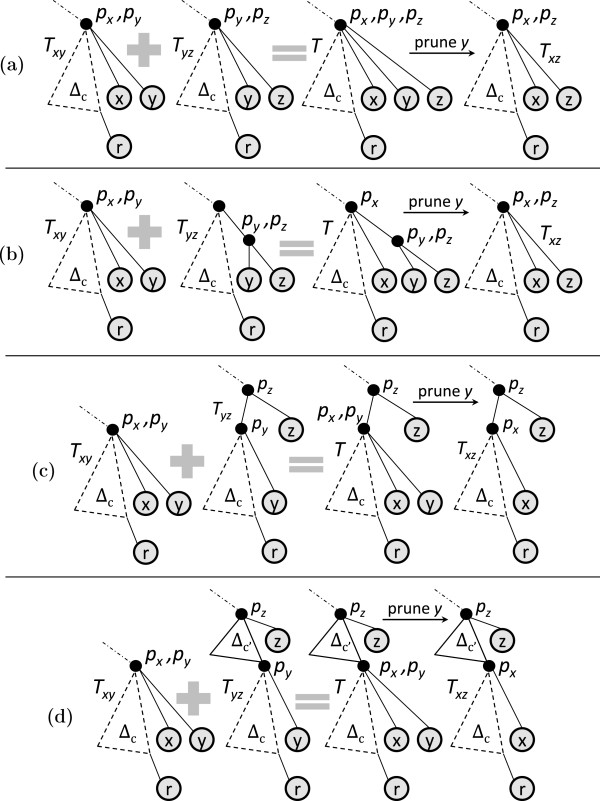
**Supportive illustrations for the proof of Lemma **1**, part 1.***T*_*xy*_ is a result of type-1 join in all the cases. **(a)***T*_*yz*_ is a result of type-1 join. **(b)***T*_*yz*_ is a result of type-2 join. **(c)***T*_*yz*_ is a result of type-3 join. **(d)***T*_*yz*_ is a result of type-4 join.

1. Suppose *T*_*x* *y*_ is a result of a type-1 join. Thus, *ψ*(*x*)<*ψ*(*y*) and agreement triplet [*r*,*x*,*y*] is of type (*r*,*x*,*y*). We have four possibilities to consider. 

(a) *T*_*y* *z*_ is a result of a type-1 join (see Figure 16(a)). Thus, *ψ*(*y*)<*ψ*(*z*) and agreement triplet [ *r*,*y*,*z*] is of type (*r*,*y*,*z*). Potential AST *T* must be obtained by grafting *z* in *T*_*x* *y*_. Since *T* must display (*r*,*y*,*z*), there is only one possibility of grafting *z* in *T*_*x* *y*_: *z* should be grafted on the common parent of *x* and *y*. Thus, AST *T* exists. Further, since, *ψ*(*x*)<*ψ*(*y*)<*ψ*(*z*), there is only one possible canonical topology for potential AST *T*: see Figure 16(a). Since *T* has *x*, *y*, *z*, as the third-to-last, second-to-last and last leaf respectively in the IDFT, pruning *y* will result in a tree that is (a) canonical, (b) has *z* as the last leaf in the IDFT, and (c) has *x* as the second-to-last leaf in the IDFT. By definition, the resulting tree is *T*_*x* *z*_ (see Figure 16(a)). Thus, *T*_*x* *z*_ exists. Further, the topology of *T*_*x* *z*_ implies that *T*_*x* *z*_ is a result of type 1 join. Similarly, pruning the last leaf, i.e., *z*, in *T* will result in *T*_*x* *y*_. Thus, $E_{T}$ is a a child of $E_{T_{\text{xy}}}$, thus, a descendant of *X*.

(b) *T*_*y* *z*_ is a result of a type-2 join (see Figure 16(b)). Thus, *ψ*(*y*)<*ψ*(*z*) and agreement triplet [ *r*,*y*,*z*] is of type (*r*,(*y*,*z*)). Potential AST *T* must be obtained by grafting *z* in *T*_*x* *y*_. Since *T* must display (*r*,(*y*,*z*)), there is only one possibility of grafting *z* in *T*_*x* *y*_: *z* should be grafted on the edge (*p*_*y*_,*y*). Thus, AST *T* exists. Further, since, *ψ*(*x*)<*ψ*(*y*)<*ψ*(*z*), there is only one possible canonical topology for potential AST *T*; see Figure 16(b). Since *T* has *x*, *y*, *z* as the third-to-last, second-to-last and last leaf respectively in the IDFT, pruning *y* will result in a tree that is (a) canonical, (b) has *z* as the last leaf in the IDFT, and (c) has *x* as the second-to-last leaf in the IDFT. By definition, the resulting tree is *T*_*x* *z*_ (see Figure 16(b)). Thus, *T*_*x* *z*_ exists. Further, the topology of *T*_*x* *z*_ implies that *T*_*x* *z*_ is a result of a type-1 join. Similarly, pruning the last leaf, i.e., *z*, in *T* will result in *T*_*x* *y*_. Thus, $E_{T}$ is a a child of $E_{T_{\text{xy}}}$, thus, a descendant of *X*.

(c) *T*_*y* *z*_ is a result of a type-3 join (see Figure 16(c)). Potential AST *T* must be obtained by grafting *x* in *T*_*y* *z*_. Since *T* must display (*r*,*x*,*y*), there is only one possibility of grafting *x* in *T*_*y* *z*_: *x* should be grafted on the parent of *y*. Thus, AST *T* exists. Further, since, *ψ*(*x*)<*ψ*(*y*), there is only one possible canonical topology for potential AST *T*; see Figure 16(c). Since *T* has *x*, *y*, *z* as the third-to-last, second-to-last and last leaf respectively in the IDFT, pruning *y* will result in a tree that is (a) canonical, (b) has *z* as the last leaf in the IDFT, and (c) has *x* as the second-to-last leaf in the IDFT. By definition, the resulting tree is *T*_*x* *z*_ (see Figure 16(c)). Thus, *T*_*x* *z*_ exists. Further, the topology of *T*_*x* *z*_ implies that *T*_*x* *z*_ is a result of a type-3 join. Similarly, pruning the last leaf, i.e., *z*, in *T* will result in *T*_*x* *y*_. Thus, $E_{T}$ is a a child of $E_{T_{\text{xy}}}$, thus, a descendant of *X*.

(d) *T*_*y* *z*_ is a result of a type-4 join (see Figure 16(d)). Potential AST *T* must be obtained by grafting *x* in *T*_*y* *z*_. Since *T* must display (*r*,*x*,*y*), there is only one possibility of grafting *x* in *T*_*y* *z*_: *x* should be grafted on the parent of *y*. Thus, AST *T* exists. Further, since, *ψ*(*x*)<*ψ*(*y*), there is only one possible canonical topology for potential AST *T*; see Figure 16(d). Since *T* has *x*, *y*, *z* as the third-to-last, second-to-last and last leaf respectively in the IDFT, pruning *y* will result in a tree that is (a) canonical, (b) has *z* as the last leaf in the IDFT, and (c) has *x* as the second-to-last leaf in the IDFT. By definition, the resulting tree is *T*_*x* *z*_ (see Figure 16(d)). Thus, *T*_*x* *z*_ exists. Further, the topology of *T*_*x* *z*_ implies that *T*_*x* *z*_ is a result of a type-4 join. Similarly, pruning the last leaf (i.e., *z*) in *T* will result in *T*_*x* *y*_. Thus, $E_{T}$ is a a child of $E_{T_{\text{xy}}}$, thus, a descendant of *X*.

Similarly, one can show that if *T*_*x* *y*_ is a result of a join of type 3 or 4, irrespective of the type of join *T*_*y* *z*_ is a result of, ASTs *T* and *T*_*x* *z*_ exist, *T*_*x* *z*_ is not a result of a type-2 join, and $E_{T}$ is a descendant of *X*.

**Figure 17:**
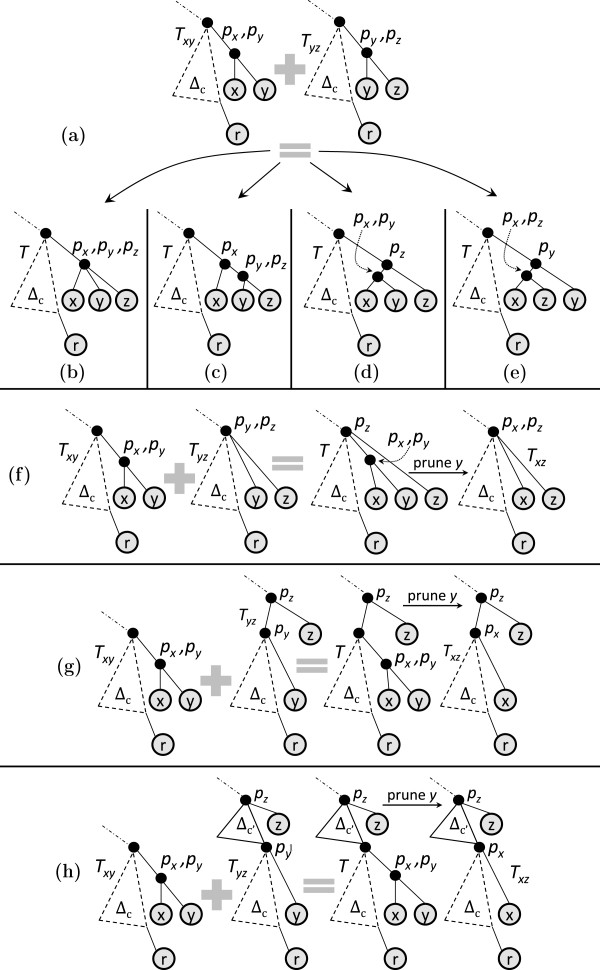
**Supportive illustrations for the proof of Lemma **1**, parts 2 and 3.***T*_*xy*_ is a result of type-2 join in all the cases. **(a)***T*_*yz*_ is a result of type-2 join. There are four possibilities for a topology that can display both *T*_*xy*_ and *T*_*yz*_: **(b)—(e)**. **(f)***T*_*yz*_ is a result of type-1 join. **(g)***T*_*yz*_ is a result of type-3 join. **(h)***T*_*yz*_ is a result of type-4 join.

2. Suppose each of *T*_*x* *y*_ and *T*_*y* *z*_ is a result of a type-2 join (see Figure 17(a)). Thus, agreement triplet [ *r*,*y*,*z*] is of type (*r*,(*y*,*z*)) and *ψ*(*y*)<*ψ*(*z*). Potential AST *T* must be obtained by grafting *z* in *T*_*x* *y*_. Since *T* must display (*r*,(*y*,*z*)) and *ψ*(*x*)<*ψ*(*y*)<*ψ*(*z*), there are four possible canonical topologies for potential AST *T*: see Figures 17(b)– 17(e). However, since [ *x*,*y*,*z*] is an agreement triplet, only one of the four topologies exists across all input trees. Thus, AST *T* exists.

Further, in all the cases discussed above, the last two leaves in the IDFT of *T* are *y* and *z* (either *y* comes before *z* or vice-versa), while *x* is the third-to-last leaf. Thus, pruning *y* in *T* will result in a canonical tree where *x* and *z* are the second-to-last and last leaves in the IDFT. By definition, the resulting tree is *T*_*x* *z*_. Thus, *T*_*x* *z*_ exists. Further, pruning the last leaf in *T* will result in either *T*_*x* *y*_ (if *z* is the last leaf in *T*) or *T*_*x* *z*_ (if *y* is the last leaf in *T*). In either case, $E_{T}$ is a descendant of *X*, as claimed.

3. Suppose *T*_*x* *y*_ is a result of a type-2 join. Thus, *ψ*(*x*)<*ψ*(*y*). Consider the following possibilities: 

(a) *T*_*y* *z*_ is a result of a type-1 join (see Figure 17(f)). Thus, *ψ*(*y*)<*ψ*(*z*). Potential AST *T* must be obtained by grafting *x* in *T*_*y* *z*_. Since *T* must display (*r*,(*x*,*y*)), there is only one possibility of grafting *x* in *T*_*y* *z*_: *x* should be grafted on the edge (*p*_*y*_,*y*). Thus, AST *T* exists. Further, since, *ψ*(*x*)<*ψ*(*y*)<*ψ*(*z*), there is only one possible canonical topology for potential AST *T*: see Figure 17(f). Since *T* has *x*, *y*, *z* as the third-to-last, second-to-last and last leaf respectively in the IDFT, pruning *y* will result in a tree that is (a) canonical, (b) has *z* as the last leaf in the IDFT, and (c) has *x* as the second-to-last leaf in the IDFT. By definition, the resulting tree is *T*_*x* *z*_ (see Figure 17(f)). Further, the topology of *T*_*x* *z*_ implies that *T*_*x* *z*_ results from a type-1 join.

(b) *T*_*y* *z*_ is a result of a type-3 join (see Figure 17(g)). Potential AST *T* must be obtained by grafting *x* in *T*_*y* *z*_. Since *T* must display (*r*,(*x*,*y*)), there is only one possibility of grafting *x* in *T*_*y* *z*_: *x* should be grafted on the edge (*p*_*y*_,*y*). Thus, AST *T* exists. Further, since, *ψ*(*x*)<*ψ*(*y*), there is only one possible canonical topology for potential AST *T*: see Figure 17(g). Since *T* has *x*, *y*, *z* as the third-to-last, second-to-last and last leaf respectively in the IDFT, pruning *y* will result in a tree that is (a) canonical, (b) has *z* as the last leaf in the IDFT, and (c) has *x* as the second-to-last leaf in the IDFT. By definition, the resulting tree is *T*_*x* *z*_ (see Figure 17(g)). Further, the topology of *T*_*x* *z*_ implies that *T*_*x* *z*_ results from a type-3 join.

(c) *T*_*y* *z*_ is a result of a type-4 join (see Figure 17(h)). Potential AST *T* must be obtained by grafting *x* in *T*_*y* *z*_. Since *T* must display (*r*,(*x*,*y*)), there is only one possibility of grafting *x* in *T*_*y* *z*_: *x* should be grafted on the edge (*p*_*y*_,*y*). Thus, AST *T* exists. Further, since, *ψ*(*x*)<*ψ*(*y*), there is only one possible canonical topology for potential AST *T*: see Figure 17(h). Since *T* has *x*, *y*, *z* as the third-to-last, second-to-last and last leaf respectively in the IDFT, pruning *y* will result in a tree that is (a) canonical, (b) has *z* as the last leaf in the IDFT, and (c) has *x* as the second-to-last leaf in the IDFT. By definition, the resulting tree is *T*_*x* *z*_ (see Figure 17(h)). Further, the topology of *T*_*x* *z*_ implies that *T*_*x* *z*_ results from a type-4 join.

Further, in all the above cases, *y* and *z* are the second-to-last and the last leaves in *T*. Thus, pruning *z* — the rightmost leaf — in *T* will result in a tree that is (a) canonical, (b) has *y* as the last leaf in the IDFT, and (c) has *x* as the second-to-last leaf in the IDFT. By definition, the resulting tree is *T*_*x* *y*_. Hence, $E_{T}$ is a descendant of $E_{T_{\text{xy}}}$, as claimed.

□

### **Proof of Lemma 2**.

Without loss of generality, let *ψ*(*y*)<*ψ*(*z*). Let *p*_*x*_, *p*_*y*_ and *p*_*z*_ denote the parent of *x*, *y* and *z* respectively. Consider the following cases: 

1. ${\text{depth}}^{T_{\text{xy}}}(p_{y})>{\text{depth}}^{T_{\text{xz}}}(p_{z})$ : As per Theorem 1, *T*_*x*−*y* *z*_ exists as a result of a type-4 join.

2. ${\text{depth}}^{T_{\text{xz}}}(p_{z})>{\text{depth}}^{T_{\text{xy}}}(p_{y})$ : As per Theorem 1, *T*_*x*−*z* *y*_ exists as a result of a type-4 join.

3. ${\text{depth}}^{T_{\text{xy}}}(p_{y})={\text{depth}}^{T_{\text{xz}}}(p_{z})$ : Consider the following sub-cases: 

(a) Agreement triplet [ *x*,*y*,*z*] is of type (*x*,*y*,*z*). Thus, depth(LCA(*x*,*y*))=depth(LCA(*y*,*z*))=depth(LCA(*x*,*z*)) across all input trees. Thus, as per Theorem 1, *T*_*x*−*y* *z*_ exists as a result of a type-1 join.

(b) Agreement triplet [ *x*,*y*,*z*] is of type (*x*,(*y*,*z*)). Thus, depth(LCA(*x*,*y*))=depth(LCA(*x*,*z*)) and depth(LCA(*x*,*y*))<depth(LCA(*y*,*z*)) across all input trees. Thus, as per Theorem 1, *T*_*x*−*y* *z*_ exist as a result of a type-2 join.

(c) Agreement triplet [ *x*,*y*,*z*] is of type ((*x*,*y*),*z*). Thus, depth(LCA(*x*,*y*))>depth(LCA(*x*,*z*)) across all input trees. Thus, as per Theorem 1, *T*_*x*−*y* *z*_ exists as a result of a type-3 join.

(d) Agreement triplet [ *x*,*y*,*z*] is of type ((*x*,*z*),*y*). Thus, depth(LCA(*x*,*z*))>depth(LCA(*x*,*y*)) across all input trees. Thus, as per Theorem 1, *T*_*x*−*z* *y*_ exists as a result of a type-3 join.

Thus, in each of the above cases, either *T*_*x*−*y* *z*_ or *T*_*x*−*z* *y*_ exists, as claimed. □

### **Proof of Lemma 3**.

*(Only If)* An AST is enumerated by joining joining two trees in an equivalence class. Thus, the union of leaf sets of trees in an equivalence class is a subset of the union of leaf sets of trees in the parent equivalence class. Extending this reasoning, the union of leaf sets of trees in an equivalence class is a subset of the union of leaf sets of trees in any ancestor equivalence class. Thus, if $E_{B}$ is a descendant of $E_{A}$, ${ℒ}_{B}$ is subset of the union of leaf sets of trees in $E_{A}$. Further, every tree in $E_{A}$ has *A* as its prefix. Thus, for every $\ell \in \{{ℒ}_{B}\setminus {ℒ}_{A}\}$, *T*_*a* *ℓ*_ must exist.

*(If)* For every $\left\{i,j\}\in \{{ℒ}_{B}\setminus {ℒ}_{A}\right\}$, *T*_*a* *i*_ and *T*_*a* *j*_ exist (*if* condition of the claim), and [ *a*,*i*,*j*] is an agreement triplet (because $\{a,i,j\}\in {ℒ}_{B}$). Thus, as per Lemma 2, either *T*_*a*−*i* *j*_ or *T*_*a*−*j* *i*_ exists. We show that there exists an $\ell \in \{{ℒ}_{B}\setminus {ℒ}_{A}\}$ such that for every $i\in \{{ℒ}_{B}\setminus \{{ℒ}_{A}\cup \ell \}\}$, *T*_*a*−*ℓ* *i*_ exists. If this is not the case, there exists a sequence of leaves $\left(\ell_{0},\ell_{1},\ldots \ell_{n})\in \{{ℒ}_{B}\setminus {ℒ}_{A}\right\}$ such that $T_{a-\ell_{0}\ell_{1}}$, $T_{a-\ell_{1}\ell_{2}}$, …, $T_{a-l_{n-1}l_{n}}$, $T_{a-l_{n}l_{0}}$ exist. Since $T_{a-l_{0}l_{1}}$ and $T_{a-l_{1}l_{2}}$ exist, and [*l*_0_,*l*_1_,*l*_2_] is an agreement triplet (because $\{l_{0},l_{1},l_{2}\}\in {ℒ}_{B}$), as per Lemma 1, AST $T_{a-l_{0},l_{2}}$ exists. Extending this reasoning, it can be shown that $T_{a-l_{0}\text{ln}}$ exists — a contradiction because $T_{x-l_{n}l_{0}}$ already exists. Thus, there exists an $\ell \in \{{ℒ}_{B}\setminus {ℒ}_{A}\}$ such that for every $i\in \{{ℒ}_{B}\setminus \{{ℒ}_{A}\cup \ell \}\}$, *T*_*a*−*ℓ* *i*_ exists. By definition, each such *T*_*a*−*l* *i*_ belongs to $E_{T_{\text{al}}}$, and $E_{T_{\text{al}}}$ is a child of *A*. Extending the same reasoning, it can be shown that there exists a sequence of ASTs (*T*_1_,*T*_2_...), where *T*_1_=*A*, such that $E_{T_{i+1}}$ is a child of $E_{i}$ and ${ℒ}_{T_{i+1}}\setminus {ℒ}_{T_{i}}$ is a leaf in $\left\{{ℒ}_{A}\setminus {ℒ}_{B}\right\}$. By definition, the last AST in the sequence is *B*. Thus, $E_{B}$ is a descendant of $E_{A}$. □

### **Proof of Lemma 4**.

Since *T*_*x* *y*_ is a result of a type-2 join, agreement triplet [ *r*,*x*,*y*] is of type (*r*,(*x*,*y*)) and *ψ*(*x*)<*ψ*(*y*). Consider any $\ell \in \{{ℒ}_{D^{c}}\setminus {ℒ}_{Y^{c}}\}$ such that [ *x*,*y*,*ℓ*] is an agreement triplet. Since *D* is a descendant of *Y*, by Lemma 3, *T*_*y* *ℓ*_ exists. Since, [ *x*,*y*,*ℓ*] is an agreement triplet, by Lemma 1, there exists an AST *T* on the leaf set ${ℒ}_{E^{c}}\cup \{x,y,\ell \}$ that displays both *T*_*x* *y*_ and *T*_*y* *ℓ*_, and $E_{T}$ is a descendant of *X*. Consider the possible topologies for *T*. (Note that we have already iterated through the possible topologies for such a *T* during the proof of Lemma 1, parts 2 and 3, but we enumerate them again for ease of reference.) Since *T*_*x* *y*_ is a result of a type-2 join, the topology of *T* depends on the type of join *T*_*y* *ℓ*_ is a result of. Consider the following cases. 

1. *T*_*y* *ℓ*_ is a result of a type-1 join. There exists only one possible canonical topology for *T*: the one corresponding to Figure 17(f) (replace *z* with *ℓ* in tree *T*).

2. *T*_*y* *ℓ*_ is a result of a type-2 join. There exists four possible canonical topologies for *T*: the ones corresponding to Figure 17(b)—17(e) (replace *z* with *ℓ* in tree *T*).

3. *T*_*y* *ℓ*_ is a result of a type-3 join. There exists only one possible canonical topology for *T*: the one corresponding to Figure 17(g) (replace *z* with *ℓ* in tree *T*).

4. *T*_*y* *ℓ*_ is a result of a type-4 join. There exists only one possible canonical topology for *T*: the one corresponding to Figure 17(h) (replace *z* with *ℓ* in tree *T*).

**Figure 18:**
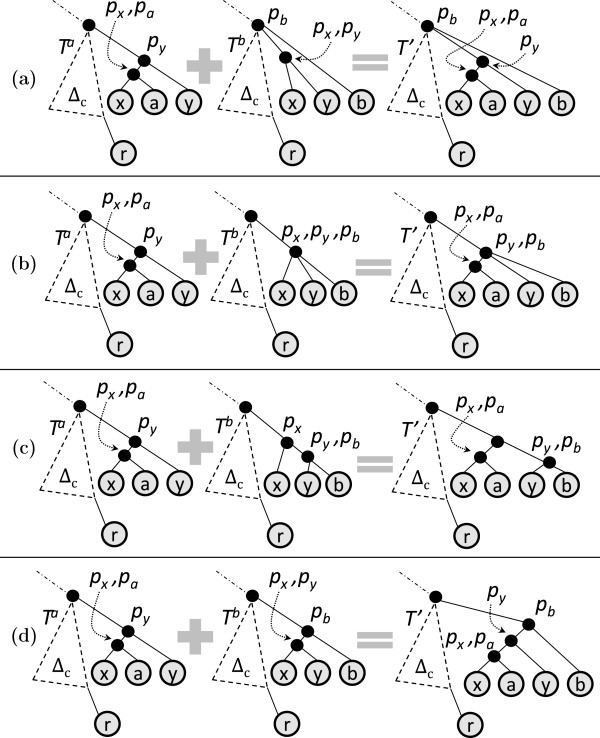
**Supportive illustrations for the proof of Lemma 4.***T*^*a*^ corresponds to the topology in Figure 17(e) (replace *z* with *a* in tree *T*) in all the cases. **(a)***T*^*b*^ corresponds to the topology in Figure 17(f) (replace *z* with *b* in tree *T*). **(b)***T*^*b*^ corresponds to the topology in Figure 17(b) (replace *z* with *b* in tree *T*). **(c)***T*^*b*^ corresponds to the topology in Figure 17(c) (replace *z* with *b* in tree *T*). **(d)***T*^*b*^ corresponds to the topology in Figure 17(d) (replace *z* with *b* in tree *T*).

**Figure 19:**
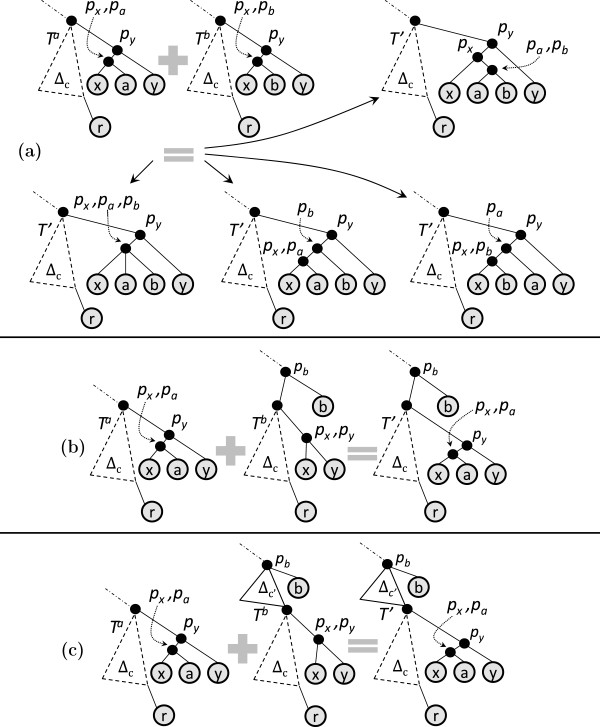
**More supportive illustrations for the proof of Lemma 4.***T*^*a*^ corresponds to the topology in Figure 17(e) (replace *z* with *a* in tree *T*) in all the cases. **(a)***T*^*b*^ corresponds to the topology in Figure 17(e) (replace *z* with *b* in tree *T*). **(b)***T*^*b*^ corresponds to the topology in Figure 17(g) (replace *z* with *b* in tree *T*). **(c)***T*^*b*^ corresponds to the topology in Figure 17(h) (replace *z* with *b* in tree *T*).

Note that out of the possible topologies for *T*, only one has *ℓ* as the second-to-last leaf in the IDFT: the one corresponding to Figure 17(e) (replace *z* with *ℓ* in tree *T*). Thus, this topology belongs to belongs to $E_{T_{\mathrm{x\ell}}}$; the rest have *y* as the second-to-last leaf in the IDFT, thus belong to $E_{T_{\text{xy}}}$. Consider any $\left\{a,b\}\in \{{ℒ}_{D^{c}}\setminus {ℒ}_{Y^{c}}\right\}$ such that [*x*,*y*,*a*] and [ *x*,*y*,*b*] are agreement triplets. Thus, by our earlier discussion in this proof, there exist ASTs *T*^*a*^ on leaf set ${ℒ}_{E^{c}}\cup \{x,y,a\}$ and *T*^*b*^ on leaf set ${ℒ}_{E^{c}}\cup \{x,y,b\}$, and, both $E_{T^{a}}$ and $E_{T^{b}}$ are descendants of *X*. Consider the following cases: 

1. Both *T*^*a*^ and *T*^*b*^ belong to $E_{T_{\text{xy}}}$. Since [ *y*,*a*,*b*] is an agreement triplet, by Lemma 2, there exists an AST on leaf set ${ℒ}_{T_{\text{xy}}}\cup \{a,b\}$. Thus, [ *x*,*a*,*b*] is an agreement triplet.

2. Both *T*^*a*^ and *T*^*b*^ do not belong to $E_{T_{\text{xy}}}$. Without loss of generality, let *T*^*a*^ belong to $E_{T_{\text{xa}}}$, i.e., it corresponds to the topology in Figure 17(e) (replace *z* with *a* in tree *T*). Thus, agreement triplet [*x*,*y*,*a*] is of type ((*x*,*a*),*y*) and *ψ*(*x*)<*ψ*(*a*). Consider potential AST *T*’ on leaf set ${ℒ}_{T_{\text{xy}}}\cup \{a,b\}$ that displays both *T*^*a*^ and *T*^*b*^. Since, the topology *T*^*a*^ is known, the topology of *T*’ depends on the topology of *T*^*b*^. Consider the following cases. In the subsequent discussion, let *p*_*x*_, *p*_*y*_, *p*_*a*_ and *p*_*b*_ denote the parent of *x*, *y*, *a* and *b* respectively. 

(a) *T*^*b*^ corresponds to the topology in Figure 17(f) (replace *z* with *b* in tree *T*). Potential AST *T*’ must be obtained by grafting *a* in *T*^*b*^. Since *T*’ must display ((*x*,*a*),*y*), there is only one possibility of grafting *a* in *T*^*b*^: *a* should be grafted on the edge (*p*_*x*_,*x*). Thus, *T*’ exists. Considering *ψ*(*x*)<*ψ*(*a*), the canonical topology for AST *T*’ is shown in Figure 18(a).

(b) *T*^*b*^ corresponds to the topology in Figure 17(b) (replace *z* with *b* in tree *T*). Potential AST *T*’ must be obtained by grafting *a* in *T*^*b*^. Since *T*’ must display ((*x*,*a*),*y*), there is only one possibility of grafting *a* in *T*^*b*^: *a* should be grafted on the edge (*p*_*x*_,*x*). Thus, *T*’ exists. Considering *ψ*(*x*)<*ψ*(*a*), the canonical topology for AST *T*’ is shown in Figure 18(b).

(c) *T*^*b*^ corresponds to the topology in Figure 17(c) (replace *z* with *b* in tree *T*). Potential AST *T*’ must be obtained by grafting *a* in *T*^*b*^. Since *T*’ must display ((*x*,*a*),*y*), there is only one possibility of grafting *a* in *T*^*b*^: *a* should be grafted on the edge (*p*_*x*_,*x*). Thus, *T*’ exists. Considering *ψ*(*x*)<*ψ*(*a*), the canonical topology for AST *T*’ is shown in Figure 18(c).

(d) *T*^*b*^ corresponds to the topology in Figure 17(d) (replace *z* with *b* in tree *T*). Potential AST *T*’ must be obtained by grafting *a* in *T*^*b*^. Since *T*’ must display ((*x*,*a*),*y*), there is only one possibility of grafting *a* in *T*^*b*^: *a* should be grafted on the edge (*p*_*x*_,*x*). Thus, *T*’ exists. Considering *ψ*(*x*)<*ψ*(*a*), the canonical topology for AST *T*’ is shown in Figure 18(d).

(e) *T*^*b*^ corresponds to the topology in Figure 17(e) (replace *z* with *b* in tree *T*). Thus, *ψ*(*x*)<*ψ*(*b*). Since, both *T*^*a*^ and *T*^*b*^ correspond to the topology in Figure 17(e), without loss of generality, let *ψ*(*a*)<*ψ*(*b*). Potential AST *T*’ must be obtained by grafting *a* in *T*^*b*^ and must display ((*x*,*a*),*y*). Since *ψ*(*x*)<*ψ*(*a*)<*ψ*(*b*), there are four possible canonical topologies for *T*’; see Figure 19(a). However, [ *y*,*a*,*b*] is an agreement triplet, thus, only one of the four possible topologies exits across all input trees Thus, *T*’ exists.

(f) *T*^*b*^ corresponds to the topology in Figure 17(g) (replace *z* with *b* in tree *T*). Potential AST *T*’ must be obtained by grafting *a* in *T*^*b*^. Since *T*’ must display ((*x*,*a*),*y*), there is only one possibility of grafting *a* in *T*^*b*^: *a* should be grafted on the edge (*p*_*x*_,*x*). Thus, *T*’ exists. Considering *ψ*(*x*)<*ψ*(*a*), the canonical topology for AST *T*’ is shown in Figure 19(b).

(g) *T*^*b*^ corresponds to the topology in Figure 17(h) (replace *z* with *b* in tree *T*). Potential AST *T*’ must be obtained by grafting *a* in *T*^*b*^. Since *T*’ must display ((*x*,*a*),*y*), there is only one possibility of grafting *a* in *T*^*b*^: *a* should be grafted on the edge (*p*_*x*_,*x*). Thus, *T*’ exists. Considering *ψ*(*x*)<*ψ*(*x*), the canonical topology for AST *T*’ is shown in Figure 19(c).

Thus, in each of the above cases *T*’ exists. Thus, [ *x*,*a*,*b*] is an agreement triplet.

This completes the proof. □

## Competing interests

The authors declare that they have no competing interests.

## Authors’ contributions

AD and DFB conceived the problem, designed the experiments and drafted the manuscript. AD designed and implemented the algorithms, and implemented the experiments. DFB coordinated the project. All authors read and approved the final manuscript.

## References

[B1] Finden C Gordon A **Obtaining common pruned trees** J Classif 1985 2 255 276 10.1007/BF01908078

[B2] Goddard W Kubicka E Kubicki G McMorris F **The agreement metric for labeled binary trees** Math Biosci 1994 123 2 215 226 10.1016/0025-5564(94)90012-4 7827420

[B3] Dong S Kraemer E **Calculation, visualization, and manipulation of, MASTs (Maximum Agreement Subtrees)** Proceedings of IEEE Computational Systems Bioinformatics Conference 2004 IEEE 405 414 10.1109/csb.2004.1332453 16448033

[B4] Farach M Thorup M **Fast comparison of evolutionary trees** Proceedings of the 5th Annual ACM-SIAM Symposium on Discrete Algorithms 1994 Philadelphia, PA, USA: Society for Industrial and Applied Mathematics 481 488

[B5] De Vienne D Giraud T Martin O **A congruence index for testing topological similarity between trees** Bioinformatics 2007 23 23 3119 3124 10.1093/bioinformatics/btm500 17933852

[B6] Lapointe F Rissler L **Congruence, consensus, and the comparative phylogeography of codistributed species in California** Am Nat 2005 166 2 290 299 10.1086/431283 16032580

[B7] Daubin V Gouy M Perrière G **A phylogenomic approach to bacterial phylogeny: evidence of a core of genes sharing a common history** Genome Res 2002 12 7 1080 1090 10.1101/gr.187002 12097345 PMC186629

[B8] Sanderson M McMahon M Steel M **Terraces in phylogenetic tree space** Science 2011 333 6041 448 10.1126/science.1206357 21680810

[B9] Bryant D **A classification of consensus methods for phylogenetics** Bioconsensus: DIMACS Working Group Meetings on Bioconsensus 2003 Amer Mathematical Society 163 163

[B10] Deepak A Fernández-Baca D Tirthapura S Sanderson M McMahon M **EvoMiner: frequent subtree mining in phylogenetic databases** Knowl Inform Syst 2013 1 32 [http://link.springer.com/article/10.1007%2Fs10115-013-0676-0]

[B11] Amir A Keselman D **Maximum agreement subtree in a set of evolutionary trees** SIAM J Comput 1994 26 758 769

[B12] Steel M Warnow T **Kaikoura tree theorems: computing the maximum agreement subtree** Inform Process Lett 1993 48 2 77 82 10.1016/0020-0190(93)90181-8

[B13] Kao M Lam T Sung W Ting H **An even faster and more unifying algorithm for comparing trees via unbalanced bipartite matchings** J Algorithms 2001 40 2 212 233 10.1006/jagm.2001.1163

[B14] Farach M Przytycka T Thorup M **On the agreement of many trees** Inform Process Lett 1995 55 6 297 301 10.1016/0020-0190(95)00110-X

[B15] Bryant D **Building trees, hunting for trees and comparing trees** PhD thesis Univ. of Canterbury, New Zealand, 1997

[B16] Huan J Wang W Prins J Yang J **Spin: mining maximal frequent subgraphs from graph databases** Proceedings of the tenth ACM SIGKDD international conference on Knowledge discovery and data mining 2004 New York, NY, USA: ACM 581 586

[B17] Thomas L Valluri S Karlapalem K **Margin: maximal frequent subgraph mining** Proceedings of the IEEE International Conference on Data Mining 2006 IEEE 1097 1101

[B18] Wang K Liu H **Discovering typical structures of documents: a road map approach** Proceedings of the 21st annual international ACM SIGIR conference on Research and development in information retrieval 1998 New York, NY, USA: ACM 146 154

[B19] Xiao Y Yao J **Efficient data mining for maximal frequent subtrees** Proceedings of IEEE International Conference on Data Mining 2003 IEEE 379 386

[B20] Chi Y Xia Y Yang Y Muntz R **Mining closed and maximal frequent subtrees from databases of labeled rooted trees** IEEE Trans Knowl Data Eng 2005 17 190 202

[B21] Zhang S Wang J **Discovering frequent agreement subtrees from phylogenetic data** IEEE Trans Knowl Data Eng 2008 20 68 82

[B22] Agrawal R Mannila H Srikant R Toivonen H Verkamo A **Fast discovery of association rules** Adv Knowl Discov Data Min 1996 12 307 328

[B23] Ramu A Kahveci T Burleigh JG **A scalable method for identifying frequent subtrees in sets of large phylogenetic trees** BMC Bioinformatics 2012 13 256 10.1186/1471-2105-13-256 23033843 PMC3543182

[B24] Margush T McMorris F **Consensus n-trees** Bull Math Biol 1981 43 239 244

[B25] Swenson K Chen E Pattengale N Sankoff D **The kernel of maximum agreement subtrees** Proceedings of International Symposium on Bioinformatics Research and Applications 2011 Springer 123 135

[B26] Pattengale N Aberer A Swenson K Stamatakis A Moret B **Uncovering hidden phylogenetic consensus in large datasets** IEEE/ACM Trans Comput Biol Bioinform 2011 8-4 99 1 10.1109/TCBB.2011.28 21301032

[B27] Guillemot S Berry V **Fixed-parameter tractability of the maximum agreement supertree problem** IEEE/ACM Trans Comput Biol Bioinform 2010 7 2 342 353 20431153 10.1109/TCBB.2008.93

[B28] Ganapathysaravanabavan G Warnow T Gascuel O, Moret B **Finding a maximum compatible tree for a bounded number of trees with bounded degree is solvable in polynomial time** Algorithms in Bioinformatics, Volume 2149 of Lecture Notes in Computer Science 2001 Berlin Heidelberg: Springer 156 163

[B29] Holland B Benthin S Lockhart P Moulton V Huber K **Using supernetworks to distinguish hybridization from lineage-sorting** BMC Evol Biol 2008 8 202 10.1186/1471-2148-8-202 18625077 PMC2500029

[B30] Lott M Spillner A Huber KT Moulton V **PADRE: a package for analyzing and displaying reticulate evolution** Bioinformatics 2009 25 9 1199 1200 10.1093/bioinformatics/btp133 19269989

[B31] Holland BR Delsuc F Moulton V Baker A **Visualizing conflicting evolutionary hypotheses in large collections of trees: using consensus networks to study the origins of placentals and hexapods** Syst Biol 2005 54 66 76 10.1080/10635150590906055 15805011

[B32] Huber KT Moulton V **Network analyses for exploring evolutionary relationships** The phylogenetic handbook: a practical approach to phylogenetic analysis and hypothesis testing Cambridge: Cambridge University Press 2009 2009

[B33] Felsenstein J Phylogenetics 2004 Sunderland, Massachusetts: Sinauer Associates

[B34] Avis D Fukuda K **Reverse search for enumeration** Discrete Appl Math 1996 65 21 46 10.1016/0166-218X(95)00026-N

[B35] Wang J Shan H Shasha D Piel W **Fast structural search in phylogenetic databases** Evol Bioinform Online 2005 1 37 46 19325851 PMC2658875

[B36] Ayres J Flannick J Gehrke J Yiu T **Sequential pattern mining using a bitmap representation** Proceedings of the eighth ACM SIGKDD international conference on Knowledge discovery and data mining 2002 New York NY, USA: ACM 429 435

[B37] Harel D Tarjan R **Fast algorithms for finding nearest common ancestors** SIAM J Comput 1984 13 338 355 10.1137/0213024

[B38] Schieber B Vishkin U **On finding lowest common ancestors: simplification and parallelization** SIAM J Comput 1988 17 1253 1262 10.1137/0217079

[B39] Bender M Farach-Colton M **The LCA problem revisited** Proceedings of the 4th Latin American Symposium on Theoretical Informatics 2000 Berlin, Heidelberg: Springer 88 94

[B40] Pattengale N Alipour M Bininda-Emonds O Moret B Stamatakis A Batzoglou S **How many bootstrap replicates are necessary?** Research in Computational Molecular Biology, Volume 5541 of Lecture Notes in Computer Science 2009 Berlin Heidelberg: Springer 184 200

[B41] Balvociute M Spillner A Moulton V **FlatNJ: A novel network-based approach to visualize evolutionary and biogeographical relationships** Syst Biol 2014 63 3 383 96 10.1093/sysbio/syu001 24436254

[B42] Huber K Moulton V **Encoding and constructing 1-nested phylogenetic networks with trinets** Algorithmica 2013 66 3 714 738 10.1007/s00453-012-9659-x

[B43] Grunewald S Spillner A Bastkowski S Bogershausen A Moulton V **SuperQ: computing supernetworks from quartets** IEEE/ACM Trans Comput Biol Bioinform 2013 10 151 160 23702551 10.1109/TCBB.2013.8

[B44] Spillner A Nguyen B Moulton V **Constructing and drawing regular planar split networks** IEEE/ACM Trans Comput Biol Bioinform 2012 9 2 395 407 21844634 10.1109/TCBB.2011.115

[B45] Huber KT Lott M Moulton V Spillner A **The complexity of deriving multi-labeled trees from bipartitions** J Comput Biol 2008 15 6 639 651 10.1089/cmb.2008.0088 18631026

[B46] Lott M Spillner A Huber K Petri A Oxelman B Moulton V **Inferring polyploid phylogenies from multiply-labeled gene trees** BMC Evol Biol 2009 9 216 10.1186/1471-2148-9-216 19715596 PMC2748082

[B47] Huber KT Moulton V Spillner A Storandt S **Computing a consensus of multilabeled trees** Proceedings of the 14th Workshop on Algorithm Engineering and Experiments 2012 Philadelphia, USA: SIAM 84 92

[B48] Czabarka ı Erdos PL Johnson V Moulton V **Generating functions for multi-labeled trees** Discrete Appl Math 2013 161 1-2 107 117 10.1016/j.dam.2012.08.010 23175592 PMC3500966

